# The infected and the affected: A longitudinal study of the impact of the COVID-19 pandemic on schoolchildren in Florida

**DOI:** 10.3389/fpubh.2023.1003923

**Published:** 2023-03-08

**Authors:** Sarah L. McKune, Daniel Acosta, Yui Fujii, Diana Joyce-Beaulieu, Md Abu Sayeed, Emilee Cato, Katelyn E. Flaherty, Ashton Creasy-Marrazzo, Ruiyu Pu, Subhashinie Kariyawasam, Anantha Arukha, Derek A. T. Cummings, Maureen T. Long, Anthony T. Maurelli, Eric J. Nelson

**Affiliations:** ^1^Department of Environmental and Global Health, University of Florida, Gainesville, FL, United States; ^2^Department of Special Education, School Psychology, and Early Childhood Studies, College of Education, University of Florida, Gainesville, FL, United States; ^3^Department of Pediatrics, University of Florida, Gainesville, FL, United States; ^4^Department of Comparative Diagnostic and Population Medicine, College of Veterinary Medicine, University of Florida, Gainesville, FL, United States; ^5^Emerging Pathogens Institute, University of Florida, Gainesville, FL, United States

**Keywords:** COVID-19, pediatrics, mental health, school-aged children, anxiety, depression, obsessive-compulsive disorder (OCD)

## Abstract

**Objectives:**

To identify risk factors associated with symptoms of anxiety, depression, and obsessive-compulsive disorder (OCD) among children during the 1st year of the COVID-19 pandemic.

**Methods:**

A longitudinal study with three cross-sectional timepoints [April 2020 (*n* = 273), October 2020 (*n* = 180), and April 2021 (*n* = 116)] was conducted at a K-12 public school in Florida. Infection and sero-positivity for SARS-CoV-2 was determined by molecular and serologic approaches. Adjusted odds ratios using mixed effect logistic regression models for symptom-derived indicators of anxiety, depression, and OCD in children in April 2021 are presented; past infection and seropositivity were included in the models.

**Results:**

The prevalence of anxiety, depression, or OCD moved from 47.1, to 57.2, to 42.2% across the three timepoints during the study. By endline of the study, in April 2021, non-white children were at higher risk for depression and OCD. Risk for anxiety, depression, and OCD was associated with students who lost a family member due to COVID-19 and who were identified as at-risk in previous timepoints. Rates of SARS-CoV-2 infection and seropositivity were low and not statistically associated with assessed outcomes.

**Conclusions:**

In situations like the COVID-19 pandemic, targeted mental health interventions and screenings are needed in children and adolescents, especially among minority children.

## Introduction

Over 476 million confirmed cases and 6.1 million deaths occurred during the first 2 years of the COVID-19 pandemic (1). While the majority of cases and deaths from infection with the etiologic agent SARS-CoV-2 have been in adults, severe and fatal cases involving children have occurred throughout the pandemic (2). Each wave of variants increased uncertainty surrounding risk of infection in children, who were largely spared infection during the first wave of COVID-19, but were increasingly susceptible to the Delta and Omicron wave of infections (3–6). Simultaneously, health related consequences of the pandemic beyond those associated with infection, including adverse mental health outcomes in children, rapidly emerged as a dominant area of interest concern (7–10). These events have led us to define two major groups: those “infected” by SARS-CoV-2 and the clinical sequalae, and those “affected” (but not “infected”) by the psychological sequalae caused by the broader impacts of the COVID-19 pandemic.

Emerging scientific literature shows that, while children constitute a small fraction of infection related morbidity and mortality (11), children worldwide have been *affected* by the pandemic in great numbers: there have been increased rates of depression, anxiety, and PTSD-related symptoms among children and adolescents since the start of the COVID-19 pandemic (12–15), with some evidence that rates of depression and anxiety have doubled since pre-pandemic levels (16). While lockdown measures and other social distancing interventions were used to protect against infection and transmission, evidence indicates that these actions may have fueled increases in negative psychosocial health (12, 17, 18). And, importantly, the effects of the pandemic on children's psychosocial health are not equally distributed. A study in Bangladesh found that children living in rural areas were less prone to suffer from mental health related problems compared to children living in urban areas (19). A systematic review of the impact of the pandemic on child and adolescent mental health showed that across the globe (inclusive of studies across Europe, Asia, Australia, North America, and South America) risk factors for adverse mental health symptoms included being female, being an adolescent, excessive exposure to COVID-19 information, previous mental health issues, community case frequency, lack of routine, and having relatives working on the front lines of COVID-19 response (20). Several other studies have also found that girls were at heightened risk for developing symptoms of anxiety and depression during the pandemic compared to boys (16, 21–23).

In the general population of the US, researchers have identified racial and ethnic disparities in mental health outcomes during the pandemic, including worsened outcomes of depression and anxiety among Black, Hispanic, and Asian adults when compared to White adults (24). A study found that those who have experienced racial discrimination in the US were at higher risk of psychological distress and increased unhealthy behaviors (e.g., smoking) than those who have not (25). Persistent systemic social inequities, the additional barriers minorities face when trying to access mental health care, disparities in food security that exacerbated during the pandemic, and the co-occurrence of racially motivated attacks (e.g., murders of minorities by the police) are also cited as reasons for the widening racial disparities in mental health outcomes observed during the pandemic (24, 26). It follows that, like in adults, the psychosocial health of children from minority groups was disproportionately negatively affected by the exacerbation of social inequities during the pandemic (27, 28). Interestingly, the multi-country systematic review did not find race or ethnicity as a risk factor for poor adolescent mental health (20). Children from racial minorities were disproportionally affected in terms of mental health outcomes, as their families are more likely to be affected by the financial and health impacts of the pandemic (29). Findings also suggest that lower socioeconomic status (SES) reported more fears about social distancing than those from higher SES, which was also the case for the subsample of Black participants in another study (30). Furthermore, children's exposure to firearm arm violence increased during the pandemic, with greater increases among children from racial minorities (31). Limited access to full-time, in-person learning as well as low social economic status, both of which unequally burden racial and ethnic minority groups, are thought to have contributed to poorer mental health outcomes in children (30, 32).

As the United States entered lockdown in March 2020, we assembled a study that aimed to identify and answer important questions at the intersection of medicine and mental health: what were the rates of viral and serologic SARS-CoV-2 positivity in school-age children? What role did these children play in household transmission? And how was the pandemic affecting their mental health? As public health professionals responding to the pandemic, this intersection and the tradeoffs between the epidemiology of infection, access to education, and equity to both education and health became a dominant point of tension. As data came in from our study and others, and rates of infection remained relatively low in children, our study aims shifted: children were suffering, but not necessarily from infection. Leveraging data collected during 12-months at the beginning of the COVID-19 pandemic (April 2020–April 2021), this paper aims to identify the children who were most *affected* by the pandemic during this time. Specifically, given what we know about the inequity in experience of the pandemic and various health outcomes outlined above, we aim to identify risk factors associated with indicators of poor mental health outcomes in school-aged children during the 1st year of the COVID-19 pandemic. The results expose high-risk groups for depression, anxiety, and OCD, underscoring which groups may benefit from targeted interventions.

## Methods

### Study context

This study took place in a K-12 developmental research school. As a developmental research school, students and their families/guardians are familiar with research activities. The school is funded by the Florida Department of Education, and it is positioned and governed by the College of Education at the University of Florida. Therefore, during the COVID-19 pandemic, the school fell under the guidance of the State University System of Florida. The school has a total student population of around 1,300 students and enrolls students according to the demographics, including race, ethnicity, gender, and income, of the state of Florida. The last day of in-person schooling during Spring of 2020 was March 13. After a 2 week extended spring break, all children were provided laptops to facilitate online learning. Students were given the option of returning to school campus in September of 2020, though the option to remain at home and connect virtually remained in place through Spring of 2021.

### Study design

The study was designed to be a prospective cohort study among students at a K-12 school to understand both patterns of transmission of SARS-CoV-2 and the psychosocial impact of the pandemic in children. However, given that student enrollment was lower than expected, and that the role of children in transmission of SARS-CoV-2 was lower than initially theorized, the study opened eligibility for enrollment to household contacts to be tested, and for students who did not initially participate to enroll at later stages of the study. Thus, the design was a longitudinal study made up of three cross-sectional timepoints. Participants were recruited from a K-12 public school in Florida. The inclusion criteria for the study limited enrollment to students at the selected school who were over 5 years old. Eligible household (HH) contacts of the students were defined as people who lived with the participant at the time of the study. There were no age restrictions for HH contacts.

**Figure 1:**
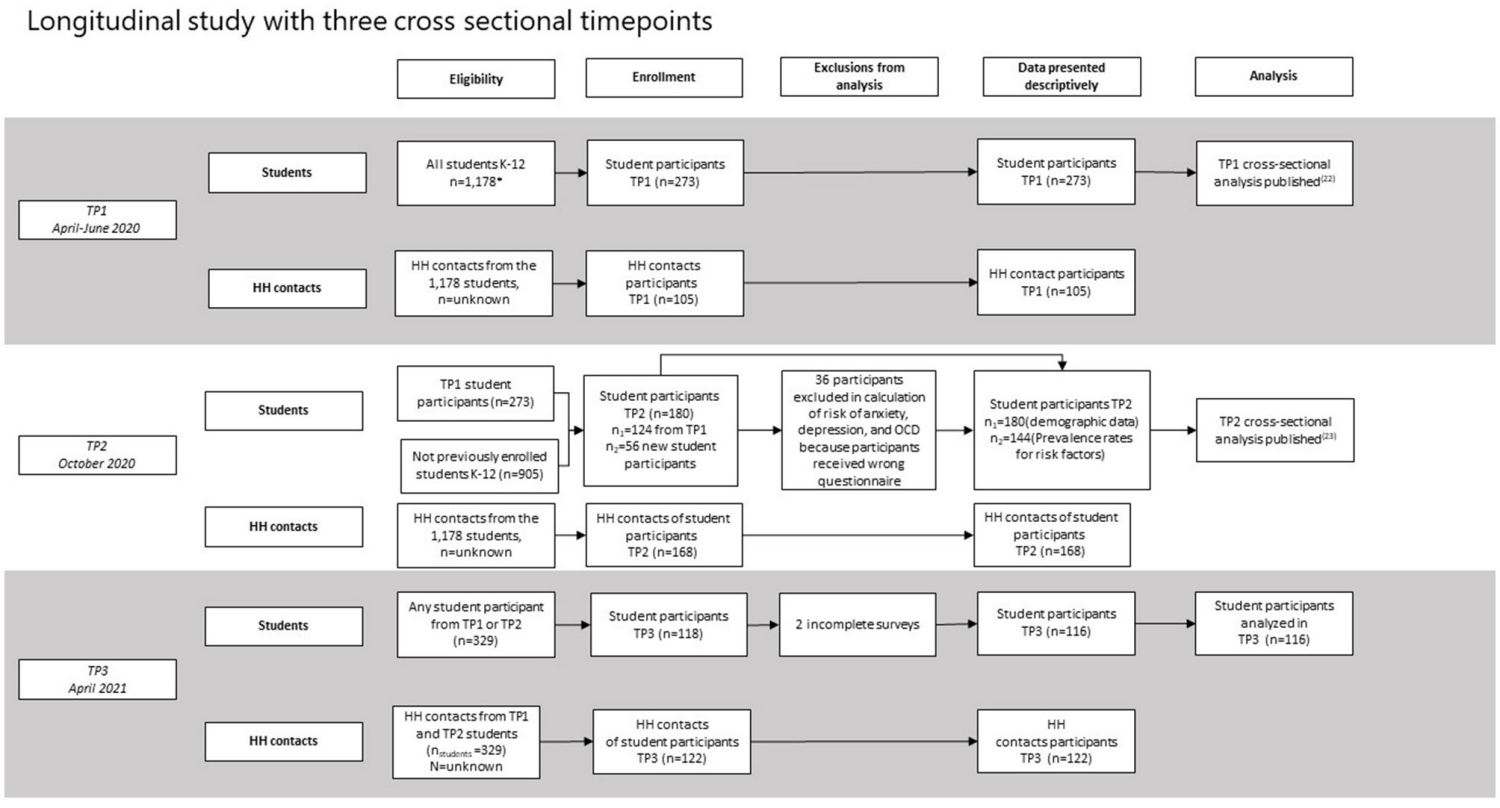
Study design, eligibility, and enrollment.

The investigators worked closely with the school administration to inform parents about the study and how they could enroll. Data were collected at three timepoints. Timepoint 1 (TP1) data were collected during April 2020; timepoint 2 (TP2) during October 2020; and timepoint 3 (TP3) during April 2021. The first round of data collection, conducted 3 weeks after all students had transitioned to a fully virtual educational format, consisted of survey data from students and their parents, and polymerase chain reaction (PCR) and antibody testing conducted solely on students. As the pandemic unfolded, and epidemiologic data indicated that infections continued to primarily affect adults, the original research protocol was modified to offer PCR and antibody testing to HH contacts of students previously enrolled; therefore, student data from April 2020 were supplemented in June with data from newly recruited household contacts; these data were combined and are treated as a single cross sectional timepoint (see Figure 1).

For TP2, students and HH contacts from TP1 as well as new participants and their HH contacts were invited to participate in an effort to increase sample size and include more underrepresented students. Finally, in TP3 all previous participants, regardless of in which timepoint they had participated, were invited to return. Enrollment of new students was not permitted during TP3, but new HH contacts from students previously participating students were allowed to enroll. Participation from students and HH contacts in the study decreased over time. The research team tried to address attrition at TP3 by increasing outreach to previous participants and including a $20 gift card as an incentive, which had not occurred during the first two timepoints of data collection.

### Sample collection and laboratory procedures

Quantitative reverse transcriptase PCR (qRT-PCR) to detect SARS-CoV-2 infection was conducted. Oral pharyngeal samples were collected by medical professionals concurrent in the period that participants completed surveys at each timepoint. The process for SARS-CoV-2 detection and quantification by rRT-qPCR have been described previously (33).

To detect previous SARS-CoV-2 infection, blood was obtained using pediatric lancets and applied to dry blood spot (DBS) cards (Whatmann Protein Saver 930). Samples were secured and transported to the BSL-2+ laboratory at the Emerging Pathogens Institute at the University of Florida for storage and processing. Serologic testing was performed to detect IgG antibody to SARS-CoV-2. Several drops of blood were collected by a fingerprick blood draw (BD Microtainer Contact-Activated Lancet) and applied to each well of a DBS card. Cards were desiccated a minimum of 72 h at room temperature. and later single 6 mm punches were taken from each blood spot and placed 66 μl of PBS-0.05% Tween and incubated with rocking at 50 rpm at 4°C overnight. After incubation, the tubes were centrifuged at 10,500 X *g* for 2 min and after incubation the supernatant removed from the paper, transferred in a new microcentrifuge tube and frozen at −80°C until use. A research ELISA targeting the Receptor Binding Domain (RBD) of the virus was used to detect exposure to SARS-CoV-2. The ELISA was adapted from a previously published protocol, which targets the RBD of the spike protein (34, 35). Briefly, 96-well ELISA plates were coated with 1 μg/ml RBD protein diluted in carbonate/bicarbonate buffer (pH 9.6) and incubated at room temperature (RT) for 1 h. Each plate was blocked with 1X Tris-buffered saline (TBS) with 5% milk and incubated for 2 h. After blocking, each sample, diluted at a concentration of 1:100 in TBS-0.5% Tween, was added in duplicate to the plate. Mouse anti-human IgG-HRP (Jackson Immunoresearch, 109-035-098) was added and incubated for 1 h. After incubation and washing, 3,3',5,5'tetramethylbensidine (TMB, Neogen Life Sciences) was added to each well, incubated for 5 min then stopped using NaSO_4_. The reaction was read using a microplate reader (Multiskan FC, Fisher or SynergyH1 BioTek) for absorbance at 450 nm. Included on each plate were positive controls consisting of a human anti-SARS-CoV-2 monoclonal antibody as well as serum from a pool of subjects who were clinically ill with COVID-19 and tested positive by rtPCR for the virus at least 4 weeks before collection of serum. A negative control consisted of a pooled serum from patients from the pre-pandemic period. Two blank wells were also included in each plate and consisted of all reagents except for primary antibody. The cut-off for this assay was based on Receiver-Operator Curve (ROC) analysis performed on 50 positive control samples and 200 prepandemic serum samples. The prepandemic samples were obtained from an archived serum bank consisting of adults that had enrolled in a diabetes study.

### Data collection instruments

Survey data were collected using REDCap as described previously in studies of TP1 and TP2 (22, 23). Questions were designed in March of 2020, when little was known about the COVID-19 pandemic. Most of the student survey instrument remained the same across the three timepoints, however, some questions were added during TP2 (23) and remained in place through TP3. These additions reflect the evolution of the pandemic (e.g., parental attitudes toward masks, lockdowns, vaccines, and parental behaviors) and are thus missing from the first round of data collection. Across the three timepoints, the student survey included questions about demographics, parental knowledge, attitudes, and practices (KAP) around COVID-19, child and adolescent symptom-derived indices for anxiety, depression, and OCD, and clinical symptoms associated with COVID-19. Demographic data collected included information such as: age, school enrollment level, parental occupation, race, ethnicity, gender, and income. Parental KAP consisted of 16 knowledge questions about COVID-19, 14 questions about attitudes toward COVID-19, and 8 questions about practices around COVID-19. From these questions, KAP scores were generated with each question counting for one point. For knowledge, all questions were considered part of the score (16 being the maximum score). For attitudes and practices, only those that were considered preventive attitudes or practices were considered for the score, with 10 and 7 being the maximum attitude and practice score, respectively. These questions, and details on how scores were developed, can be found in the Supplementary material. Risk of anxiety, depression, and OCD were assessed using a symptom-derived index created by the investigators in collaboration with a team of school psychologists associated with the study school. A 5-point Likert scale was used to assess the prevalence of symptoms. Using language appropriate for different age groups, questions were divided into two groups, those for children aged 13 years and younger and those for children older than 13 years. The associated scores to each question were also age specific; for instance, children 13 and under had the following response options: Never (1), A little (2), Sometimes (3), A lot (4), or Always/constantly (5), while adolescents over 13 had the following options: Never (1), Occasionally (2), Half the Time (3), Often (4), Always (5) (22). The questions aimed to identify the prevalence symptoms associated with anxiety, depression, and OCD. Questions included psychosomatic symptoms (e.g., fatigue, insomnia), as well as psychological symptoms (e.g., feeling nervous, irritability). If a participant responded to one or more of the questions with 3 or higher in the 5-point Likert scale (“Sometimes” for children 13 and younger and “Half the time” for adolescents over 13) they were coded “At Risk” of one of the three outcomes. While the full methodology used to develop the symptom-based psychosocial outcomes has been described elsewhere (22, 23), a detailed table showing the items used in each outcome is included in the Supplementary material and remained unchanged across the three timepoints. Psychosocial variables were only assessed for student participants, as HH contacts received a different questionnaire (the household contact survey only collected demographic information and vaccination status) which is not included in the analysis and can be found in the Supplementary material.

### Statistical analyses

Data were examined using a bivariate analysis between the outcome measures at TP3 (risk of anxiety, depression, or OCD) and the variables of interest: previous risk of anxiety, depression, or OCD at enrollment; parental vaccine attitudes; parental occupation; parental loss of income; parental risk behaviors; mode of schooling (remote or in-person); participation in sports; participation in the school band; knowledge scores; attitude scores; practice scores; parental healthy days; parental resilience scores; parental optimism scores; COVID-19 infection in household; death of a family member due to COVID-19; and household income. The variable *Previous risk of anxiety, depression, or OCD at enrollment* was a binary variable where participants had either no previous risk, or presented risk for any of the three outcomes, at the time of their enrollment in the study (TP1 or TP2). The COVID-19 infection in household variable was assessed using the answers from the survey where parents self-reported whether someone in their household had had a confirmed or suspected case of COVID-19 since the start of the pandemic. The vaccine attitude items (5-point Likert Scale, see Supplementary material) were dichotomized (Concern/No Concern and Agree/Disagree) for analysis purposes. The other variables, such as Knowledge Scores, Attitude Scores, and Practices Scores were calculated by summing correct (knowledge) or preventative (attitudes and practices) items of each category. A detailed account of the creation of these scores, and other variables has been previously published (23). Mixed effect logistic regression models were developed using only those variables that were significantly associated (*p* > 0.2) with the outcome measure in the bivariate analysis, with the exception of a few confounders of interest (race, sex, school level, COVID-19 infection in household, and death of family member due to COVID-19) which were included in the models regardless of the results of bivariate tests for significance. Using these variables, models with the lowest Akaike Information Criterion were kept for each of the dependent variables. For the variable Race, participants were given several options and a fill-in option for race, and ethnicity was asked as a separate question (see Supplementary material). However, these data were categorized into four groups Black, Hispanic, Multiracial/Other, White, in order to reflect groupings required by the State of Florida when the school reports data. These four categories are used to present data descriptively. However, for the regression models, due to the small sample size and underrepresentation of minorities in the third timepoint (one group had <5 participants), the variable Race was recoded (White/Non-White). Data on Race and the three outcome variables (Anxiety, Depression, OCD) is presented descriptively in the results. Only data from completed questionnaires were included in the analysis. Questionnaires that were partially answered or had missing values were removed from the data set.

### Ethics

All participants provided informed written digital consent/assent online (*via* REDCap) to participate in the study; assent was required for participants between 8 and 18 years of age. The study was approved by the Internal Review Board at the University of Florida.

## Results

Participant enrollment was as follows: TP1 (04/17/2020 to 06/06/2020) 273 children and 105 household contacts; TP2 (10/05/2020 to 10/17/2020) 180 children and 168 household contacts; TP3 (04/05/2021 to 04/22/2021) 116 children and 122 household contacts. Student population characteristics are shown in Table 1, while HH contact characteristics can be found in the Supplementary material. For TP3, the distribution of students by school level was similar to that of previous timepoints, roughly reflecting the distribution of the school population's age. For race, the sample from TP3 was less diverse, with 68.1% of the sample being White (an increase from 56.1 in TP2, and 62.4% in TP1). Most notably, a decrease in the participation of those categorized as Multiracial/Other was observed, from 11.1 in TP2 to 3.4% in TP3. The percentage of male participants also decreased compared to previous timepoints (48.2% in TP1 and 47.2% in TP2), to 43.1%. Table 1 also shows the prevalence of children at risk for each outcome at each of the three timepoints. These data indicate that 47.1% of students presented with symptoms consistent with OCD, anxiety, or depression in April 2020, increased to 57.2% in October 2020, and decreased to 42.2% in April 2021.

**Table 1:** Participant characteristics.

**Students**	**TP1 *n* = 273^a^**	**TP2 *n* = 180^a^**	**TP3** ***n*** = **116**^**a**^		
**School level**					
Elementary	85 (31.1%)	53 (29.4%)	28 (24.1%)		
Middle	81 (29.7%)	54 (30%)	36 (31%)		
High	107 (39.2%)	73 (40.6%)	52 (44.8%)		
**Race**					
Black	24 (8.8%)	21 (11.7%)	12 (10.3%)		
Hispanic	52 (19%)	38 (21.1%)	21 (18.1%)		
Multiracial	26 (9.5%)	20 (11.1%)	4 (3.4%)		
White	171 (62.6%)	101 (56.1%)	79 (68.1%)		
**Sex**					
Female	142 (52%)	95 (52.8%)	66 (56.9%)		
Male	131 (48%)	85 (47.2%)	50 (43.1%)		
**Prevalence of risk**	**TP1** ***n*** = **273**	**TP2** ***n*** = **144**^b^	**TP3** = **116**		
Anxiety	34.3%	42.4%	34.5%		
Depression	35.8%	45.1%	27.6%		
OCD	32.8%	41.7%	31.9%		
Anxiety, depression, or OCD	47.1%	57.2%	42.2%		
**PCR**	**TP1** ***n*** = **265**	**TP2** ***n*** = **146**	**TP3** ***n*** = **102**		
Positive	0 (0%)	2 (1.4%)	0 (0%)		
Negative	265 (100%)	144 (98.6%)	102 (100%)		
**Serology**	**TP1** ***n*** = **252**	**TP2** ***n*** = **130**	**TP3** ***n*** = **99**		
			Fully vaccinated *n* = 7	Partially vaccinated *n* = 14	Not vaccinated *n* = 78
Positive	1 (0.4%)	5 (3.8%)	7 (100.0%)	10 (71.4%)	5 (6.4%)
Negative	250 (99.2%)	125 (96.2%)	0 (0.0%)	4 (28.6%)	71 (91.0%)

a TP1, April 2020; TP2, October 2020; TP3, April 2021. Only completed surveys are included in the analysis.

b For TP2 the sample size decreased to *n* = 144 for variables regarding the risk of anxiety, depression, and OCD as 36 participants were taken out of the analysis as they received the wrong psychosocial questionnaire due to a technical issue.

At TP1, all student participants tested were negative by PCR and one (0.4%) was IgG positive for SARS-CoV-2. All household contacts tested were negative by PCR and one (1.1%) was IgG positive for SARS-CoV-2. At TP2, two students (1.4%) tested positive by PCR and five students (3.8%) were IgG positive for SARS-CoV-2. All household contacts tested negative by PCR and six household contacts (3.9%) were IgG positive for SARS-CoV-2. At TP3, all students tested negative by PCR and 22 students (22.2%) were IgG positive for SARS-CoV-2. Seven students were fully vaccinated and 14 were partially vaccinated (only had received 1 dose of a 2-dose series vaccine against COVID-19). All fully vaccinated students and 10 of the partially vaccinated (71.4%) were IgG positive for SARS-CoV-2. All household contacts tested negative by PCR and 88 (83.8%) were IgG positive for SARS-CoV-2. Sixty-five household contacts were fully vaccinated, from which 62 (95.4%) were IgG positive for SARS-CoV-2. Twenty-seven household contacts were partially vaccinated, from which 24 (88.9%) were IgG positive for SARS-CoV-2.

The analysis of anxiety, depression, and OCD risk factors at TP3 used regression models that adjusted for multiple factors (Table 2). Race was significantly associated with risk of depression and OCD, with Non-White participants being at higher risk of depression [aOR = 3.45, CI 95% = (1.13–10.58)], and OCD [aOR = 4.51, CI 95% = (1.52–13.34)] compared to White participants. Death of a family member due to COVID-19 was associated with higher risk of anxiety [aOR = 6.49, CI 95% = (1.00–42.08)] and depression [aOR = 25.23, CI 95% = (2.34–271.58)]. There was no significant difference in risk for anxiety, depression, and OCD between males and females. For school level, being a high school student was associated with higher risk of anxiety [aOR = 6.97, CI 95% = (1.68–28.95)]. Being previously identified as at risk during TP1 or TP2 for any of the three outcomes of the study was also associated with risk of anxiety [aOR = 10.8, CI 95% = (3.03–38.47)], depression [aOR = 5.42, CI 95% = (1.52–19.36)], and OCD [aOR = 14.67, CI 95% = (3.8–56.65)] in TP3. Higher parental COVID-19 knowledge scores were a significant protective factor against risk of depression [aOR = 0.56, CI 95% = (0.35–0.89)]. Higher parental COVID-19 attitude scores were associated with risk of anxiety [aOR = 1.51 CI 95% = (1.03–2.23)]. Parental belief that vaccinating their child was not important for the health of others in the community was protective against their child presenting at risk for OCD [aOR = 0.16 CI 95% = (0.03–0.92)]. No statistically significant associations were found between risk (anxiety, depression, and/or OCD) and additional variables included in the analyses, including COVID-19 household infection.

**Table 2:** Determinants of anxiety, depression, and OCD at timepoint 3 (TP3).

**Variables**	**Anxiety: aOR^a^ (95%CI)**	**Depression: aOR^a^ (95%CI)**	**OCD: aOR^a^ (95%CI)**
**Race**			
White (Reference)	Ref	Ref	Ref
Non-White^b^	2.58 (0.89–7.5)	**3.45 (1.13**–**10.58)**	**4.51 (1.52**–**13.34)**
**Sex**			
Male (Reference)	Ref	Ref	
Female	0.69 (0.26–1.89)	2.7 (0.85–8.59)	0.68 (0.25–1.91)
**School level**			
Primary (Reference)	Ref	Ref	Ref
Middle School (2)	3.46 (0.92–13.07)	3.41 (0.76–15.32)	1.64 (0.44–6.15)
High School (1)	**6.97 (1.68**–**28.95)**	2.3 (0.5–10.53)	3.02 (0.74–12.31)
**Prior risk**			
Previous risk of anxiety, depression, OCD	10.8 (3.03–38.47)	5.42 (1.52–19.36)	14.67 (3.8–56.65)
**Exposure and deaths in family**			
Death in Family due to COVID−19	**6.49 (1.00**–**42.08)**	25.23 (2.34–271.58)	1.77 (0.33–9.41)
COVID-19 household infection	2.01 (0.47–8.48)	0.57 (0.12–2.85)	1.92 (0.47–7.82)
**Parental COVID-19 Vaccine attitudes and parental knowledge, attitudes, and practices (KAP) around COVID-19**			
Ability to openly discuss COVID-19 vaccine with child's doctor [Binary variable, Agree(Ref)/Don't Agree]	0.17 (0.02–1.75)	NA	NA
Parental perception of the importance of COVID-19 vaccines for the health of the community [Binary variable, Agree (Ref) /Don't Agree]	NA	NA	**0.16 (0.03**–**0.92)**
Knowledge Index Score (0–16), with 0 being low knowledge and 16 being high knowledge.	NA	**0.56 (0.35**–**0.89)**	NA
Attitude Score (0–10) with 0 being lowest protective attitude and 10 being highest protective attitude score	**1.51 (1.03**–**2.23)**	NA	NA
Practice Score (0–8) with 0 being lowest protective practice and 8 being highest protective practice score	NA	NA	1.35 (0.88–2.08)
**Participation in sports**			
Child participating in Sports during the Spring	NA	NA	2.34 (0.67–8.14)

a aOR, Adjusted Odds Ratios. Bold text designates 95% confidence intervals above or below aOR of 1. NA, Not applicable, as not all variables were used in each model.

b Disaggregated race data could not be include in the analysis due to small sample size.

Race, coded as Black, White, Multiracial/Other, or Hispanic, could not be included in the model due to a small sample size. The prevalence of White students presenting at risk for anxiety was 27.8%, for depression 20.3%, and for OCD 22.8%; this was lower than for Black participants at 50.0, 41.7, and 50.0%, Hispanic participants at 47.6, 47.6, and 42.9%, and Multiracial/Other participants at 50.0, 25.0, and 100%, respectively.

## Discussion

Children are at less risk of serious clinical complications from SARS-CoV-2. However, even though our sample population had low rates of infection at each time point (consistent with positivity rate trends within the community), symptoms associated with risk for anxiety, depression, and OCD were persistent across the study period. At least 42.2% of the participants presented as at risk for depression, anxiety, or OCD throughout the study period. The prevalence of symptoms peaked during TP2. During TP1 and TP2, race was not significantly associated with risk of anxiety, depression, or OCD (22, 23). For TP3, however, when the prevalence of students at risk was lowest, a significant association between race and risk for depression and OCD emerged, with minorities being at higher risk.

The association between race and mental health or psychosocial outcomes has emerged as a prominent feature of the COVID-19 pandemic (28, 32). Our prior studies at TP1 and TP2 did not find a link between race and mental health, however the association was prominent at TP3. One explanation is that rates were similar early yet differed at TP3 because racial minorities might experience slower rates of mental health recovery after the initial shock at TP1 and TP2 that nearly all children experienced. This finding on race is also accompanied by a significant longitudinal association of anxiety, depression, and OCD at TP1 and subsequent risk at TP3. This could also be because of progressive negative effects of the pandemic (both health-related and social issues) have disproportionately affected minorities (36–38). During the COVID-19 pandemic, research found profound racial and ethnic inequalities in schools when it comes to access to resources and engagement in school (32, 39). These disparities across studies may reflect the temporally dynamic nature of the psychosocial impact of the COVID-19 pandemic. They also highlight how the pandemic could have an amplified effects on minorities, even if not infected they are likely to be more affected due to the cumulative effect of different factors that have impacted minorities at a greater scale

Grade level was associated with risk of anxiety, with students from High School being at highest risk. Previously published analyses on this study population found that elementary school students were at higher risk for poor psychosocial outcomes, thus suggesting a recovery of younger students that did not occur to the same degree among older students. A study in Greece, which focused on senior High School students, found that there was an increase in anxiety and depressive symptoms across this population, with girls and those reporting symptoms at baseline being at higher risk (12). This might suggest that students in elementary and middle school are less likely to remain at risk than high school students. Moreover, there was a strong association with symptoms of anxiety and depression in participants who had lost a family member due to COVID-19, which is a concerning association that other studies have found (10, 40, 41). Given the disproportionate mortality among Black and other minority communities, this may explain the association with non-white populations seen above.

Regarding KAP, parental attitudes supportive of protective measures against COVID-19 were associated with children presenting symptoms of anxiety. High protective attitude scores could be related to higher levels of parental fear toward COVID-19, which has been associated with parents being less concerned with protecting children's mental health (42). Conversely, parental knowledge of COVID-19 was a protective factor for depressive symptoms, which is congruent with findings from other studies (43, 44). When it came to COVID-19 vaccine attitudes, children whose parents indicated that COVID-19 vaccines were not important for the health of the community were less likely to present with symptoms of OCD. While COVID-19 related KAP should serve to protect against COVID-19—and it may have—the same knowledge, attitudes, and practices may exacerbate *other* health outcomes, including anxiety, depression, and OCD. These findings, on the associations between parental COVID-19 related KAP and children's psychosocial outcomes are complex. Previously published data from this study population (TP2) align with this point, as parental knowledge scores were strongly predictive of anxiety and protective attitudes by parents were associated with symptoms of OCD in children (23). This variation in the role of KAP, where some present as protective and others as risk factors, highlight the importance and complexity of parental KAP as a driver for children's health.

## Limitations

Given the urgency to launch the study as the COVID-19 pandemic began in the state of Florida in 2020, a sample size calculation was not performed *a priori*. Sample sizes were limited at each timepoint, especially for TP3 (*n* = 116), which may have resulted in an enrollment bias. In addition, the small sample size may affect interpretability of results. As a developmental research school, the demographics of the school are designed to match those of the state. Compared to the school population, minority students and families were underrepresented in our study and wealthier students were overrepresented (reported median income of participating households was $100,000 USD). The surveys were also only available in English, which could have limited participation of families were English is not the first language. These factors could limit the generalizability of the study, particularly for low-income, rural, and minority populations not represented in the study. While the study was a prospective longitudinal study, not all participants participated at all time points. This likely results in heterogeneity and reduced statistically significant effect sizes.

## Conclusions

The findings of this study highlight the need for mental health screening and support for school-aged children during a pandemic. Targeted interventions should also be implemented, with a strong focus on addressing the racial disparities in access to mental health resources, as our research suggests that minorities are at higher risks of presenting symptoms associated with depression and OCD 12 months into the pandemic.

## Data availability statement

The original contributions presented in the study are included in the article/Supplementary material, further inquiries can be directed to the corresponding author.

## Ethics statement

The University of Florida's Internal Review Board approved this study (IRB202001345), which was conducted under a parent study (IRB202000488). Written informed consent/assent, as appropriate, was provided.

## Author contributions

SM led the study. SM, EN, and AM conceived, designed, and supervised the study and assisted with data analysis and drafting of the manuscript. DA and YF led the analysis of the data collected *via* surveys, as well as led the drafting of the manuscript. DJ-B led the design and interpretation of the psychosocial variables and guided the analysis and interpretation of the data. EN led the laboratory aspect of the study. EC, KF, AC-M, RP, SK, AA, DC, and ML conducted the laboratory data analysis and collaborated in the design of the laboratory aspect of the study. All authors contributed to the article and approved the submitted version.
