# Supplementary material for: The infected and the affected: A longitudinal study of the impact of the COVID-19 pandemic on schoolchildren in Florida
Source: Front Public Health. 2023 Mar 8;11:1003923. doi: 10.3389/fpubh.2023.1003923 (PMC10030597; doi:10.3389/fpubh.2023.1003923)
Supplement: Supplementary file 1 [file Table_1.docx]

**METADATA**

Contents

[1. Data set descriptors 1](#_Toc105423685)

[2. Research Descriptors 2](#_Toc105423686)

[3. Data Set Status and Accessibility 2](#_Toc105423687)

[4.1 Variables 2](#_Toc105423688)

[Baseline demographics/Data Processing Variable 3](#_Toc105423689)

[Laboratory Results 3](#_Toc105423690)

[TP1 Questionnaire 4](#_Toc105423691)

[TP2 Questionnaire 21](#_Toc105423692)

[TP3 Questionnaire 53](#_Toc105423693)

[HH Contact Questions (TP3) 55](#_Toc105423694)

[5.Supplemental Descriptors 59](#_Toc105423695)

# 1. Data set descriptors

**Data set identity**

Survey data from parents and students K-12 school. Data contains self-reported symptoms for symptoms associated with depression, anxiety, and obsessive compulsive disorder (OCD), as well as knowledge, attitudes, and practices around COVID-19. Data also contain COVID-19 test results of participants (antibodies and PCR tests).

**Investigators:**

The principal investigators of the study are Dr. Sarah L. McKune, Dr. Eric J Nelson, and Dr. Anthony T. Maurelli. For any questions or concerns about the data set, please contact Dr. Sarah McKune (smckune@ufl.edu) or Daniel Acosta (daniel.acosta@ufl.edu).

**Funding:**

This research was supported by the University of Florida Clinical and Translational Science Institute, which is supported in part by the NIH National Center for Advancing Translational Sciences under award number UL1TR001427. The content is solely the responsibility of the authors and does not necessarily represent the official views of the National Institutes of Health. The research was also supported by the University of Florida College of Public Health and Health Professions, the College of Medicine, the Emerging Pathogens Institute at the University of Florida, and the Florida Children’s Miracle Network. The costs of publication of this article were supported by the University of Florida Emerging Pathogens Institute.

**Research Objectives**

Understand the role of parental knowledge attitudes and practices, as well as infection status of household, and other sociodemographic characteristics, in the risk of children presenting symptoms for depression, anxiety, and OCD.

Keywords: COVID-19; students; psychosocial health; virtual learning; depression; anxiety; OCD; environment

# 2. Research Descriptors

**Timeframe and location**

Data was collected in from April of 2020 to April of 2021 in a public school in Gainesville, Florida, United States.

**Research Methods**

Survey data was collected using online surveys connected to the HIPPA compliant interface REDCap. Clinical samples were collected by one of the principal investigators (Dr. Eric Nelson) with other volunteers from medical fields (nurses, doctors). Laboratory samples were processed by the Emerging Pathogens Institute at the University of Florida under the supervision of Dr. Eric Nelson. Data were collected from students of the selected K-12 school and members of their household.

Data collection was managed by the Clinical and Translational Science Institute at the University of Florida, and by graduate research assistant Daniel Acosta. The study was conducted according to the guidelines of the Declaration of Helsinki, and approved by the University of Florida Institutional Review Board, protocol IRB202001345

# 3. Data Set Status and Accessibility

Please email Dr. Sarah McKune ([smckune@ufl.edu](mailto:smckune@ufl.edu)) to obtain a de-identified data set.

**Information on data set structure, organization, and how values are to be interpreted**

Data is in 1 single .csv file that has 549 columns and 559 rows. The data codebook is presented below.

**IMPORTANT TIEMPOINT CONSIDERATION**

The study has 3 timepoints referred to as TP1, TP2, and TP3. However, TP1 had two instances of data collection. One in April of 2020 only for students and one in June 2020, where students and HH_contacts were invited to participate. Data collected in this period is labeled with tp1, however, for laboratory data, to differentiate the samples collected in April and June, the ones from April are labeled tp1, and the ones from June are labeled tp1.5.

TP2 refers to data collected in October of 2020. TP3 refers to data collected in April of 2021.

## 4.1 Variables

Below is the list of all the variables listed in the .csv file. The variable name, a description of the variable or the questions as asked to participants, and the possible values/range are shown below. All missing values appear as “NA”.

### Baseline demographics/Data Processing Variable

record_id: numeric variable with a unique identifier for each participant (possible range 0-999).

type_of_participant: Defines the type of participant, possible values: Student, HH_contact

tp1_crossection: Binary variable indicating if the participant participated in the study in timepoint 1 (April/June 2020). Values: 1=Participated, 0=Did not participate.

tp2_crossection: Binary variable indicating if the participant participated in the study in timepoint 2 (October 2020). Values: 1=Participated, 0=Did not participate.

tp3_crossection: Binary variable indicating if the participant participated in the study in timepoint 3 (April 2021). Values: 1=Participated, 0=Did not participate.

hh_id: numeric variable with a unique identifier for each household (possible range 0-999).

sentinel_child: Binary variable indicating the first child of a household to sign up for the study. This variable was not used for analysis, but it was important in generating the family clusters.

race_recoded: Race of participants recoded to match the reporting used by the state of Florida. The possible values are: Black, Hispanic, Multiracial/Other, White.

sex: Sex of the participant. Possible values are Male/Female.

student_dob: The student’s date of birth in the MM/DD/YYYY format.

student_reported_age_enrollment: The age of the student was enrolled in at time of consenting to participate in the study (either parental consent, or student consent for students 18 and older). Range of possible values=5-18.

student_grade= The school grade the student was enrolled in at time of consenting to participate in the study (either parental consent, or student consent for students 18 and older). Possible values 0-12, where 0=K, 1= First grade, 2= Second grade, and so on.

enrollment_school_level: School level at which the participant enrolled. Possible values: Primary School, Middle School, High School, NA (for household contacts).

tp2_tp3_school_level: School level of participants in tp2 and tp3. In some instances, students enrolled in tp1 changed school levels. Possible values: Primary School, Middle School, High School, NA (for household contacts).

tp1_income= household income reported at TP1 (not reported by all participants).

### Laboratory Results

tp1_student_covid_pcr_result= COVID-19 PCR results for students for TP1. Possible values (0=Negative, 1=Positive).

tp1_student_igg_antibodies= COVID-19 antibody (IgG) results for students for TP1. Possible values (0=Negative, 1=Positive, 2= Intermediate, 99=Inadequate).

tp1.5_student_covid_pcr_result= COVID-19 PCR results for students for TP1.5. Possible values (0=Negative, 1=Positive).

tp1.5_student_igg_antibodies= COVID-19 antibody (IgG) results for students for TP1.5. Possible values (0=Negative, 1=Positive, 2= Intermediate, 99=Inadequate).

tp1.5_hhc_covid_pcr_result= COVID-19 PCR results for household contacts for TP1.5. Possible values (0=Negative, 1=Positive).

tp1.5_hhc_igg_antibodies= COVID-19 antibody (IgG) results for household contacts for TP1.5. Possible values (0=Negative, 1=Positive, 2= Intermediate, 99=Inadequate).

tp2_student_covid_pcr_result: OVID-19 PCR results for students for TP2. Possible values (0=Negative, 1=Positive).

tp2_student_igg_antibodies: COVID-19 antibody (IgG) results for students for TP2. Possible values (0=Negative, 1=Positive, 2= Intermediate, 99=Inadequate

tp2_hhc_covid_pcr_result: OVID-19 PCR results for students for TP2. Possible values (0=Negative, 1=Positive).

tp2_hhc_igg_antibodies: COVID-19 antibody (IgG) results for students for TP2. Possible values (0=Negative, 1=Positive, 2= Intermediate, 99=Inadequate

tp3_student_covid_pcr_result: OVID-19 PCR results for students for TP3. Possible values (0=Negative, 1=Positive).

tp3_student_igg_antibodies: COVID-19 antibody (IgG) results for students for TP3. Possible values (0=Negative, 1=Positive, 2= Intermediate, 99=Inadequate

tp3_hhc_covid_pcr_result: OVID-19 PCR results for students for TP3. Possible values (0=Negative, 1=Positive).

Tp3_hhc_igg_antibodies: COVID-19 antibody (IgG) results for students for TP3. Possible values (0=Negative, 1=Positive, 2= Intermediate, 99=Inadequate

tp3student_covid_vaccine_status: Variable to assess if student was vaccinated for COVID-19 at tp3. Two possible values: Half Dose= Partially vaccinated for COVID-19 (1 out of a 2 dose series, or not yet 14 days after second dose) and Full Dose=Fully vaccinated for COVID-19 (14 days or more after the second dose of a 2-series COVID-19 vaccine).

*Note*: Household contact vaccine status is located with the TP3 information for HH contacts.

### TP1 Questionnaire

**For the variables below, three elements will be shown. 1. The name of the variable, 2. The question participants got, and 3. The possible answers and the code. All response options were given to participants as radio buttons, unless specified otherwise. Participants answering questions in this sections are the parents or guardians of students.**

| Variable name | Question asked participants | Possible answers/Code |
| --- | --- | --- |
| parental_years_schooling | How many years of school did you complete? | text (number) |
| Variable name | Question asked participants | Possible answers/Code |
| tp1_k1 | K1. The main clinical symptoms of COVID-19 are fever, fatigue, dry cough, and muscle aches. | 1, True \| 0, False \| 2, Don't know |
| tp1_k2 | K2. Unlike the common cold, stuffy nose, runny nose, and sneezing are less common in persons infected with the COVID-19 virus. | 1, True \| 0, False \| 2, Don't know |
| tp1_k3 | K3. There currently is no effective cure for COVID-19, but early symptomatic and supportive treatment can help most patients recover from the infection. | 1, True \| 0, False \| 2, Don't know |
| tp1_k4 | K4. Antibiotics can be used to treat COVID-19. | 1, True \| 0, False \| 2, Don't know |
| tp1_k5 | K5. Not all persons with COVID-19 will develop to severe cases. | 1, True \| 0, False \| 2, Don't know |
| tp1_k6 | K6. People of all racial and ethnic groups can become infected with the COVID-19 virus. | 1, True \| 0, False \| 2, Don't know |
| tp1_k7 | K7. Most people who are infected with the COVID-19 virus recover from it. | 1, True \| 0, False \| 2, Don't know |
| tp1_k8 | K8. Handwashing can help reduce transmission of the COVID-19 virus. | 1, True \| 0, False \| 2, Don't know |
| tp1_k9 | K9. Persons with COVID-19 cannot pass the virus to others if they do not have symptoms. | 1, True \| 0, False \| 2, Don't know |
| tp1_k10 | K10. The COVID-19 virus spreads via respiratory droplets of infected individuals. | 1, True \| 0, False \| 2, Don't know |
| tp1_k11 | K11. Ordinary residents can wear general medical masks to prevent infection by the COVID-19 virus. | 1, True \| 0, False \| 2, Don't know |
| tp1_k12 | K12. It is not necessary for children and young adults to take measures to prevent infection by the COVID-19 virus. | 1, True \| 0, False \| 2, Don't know |
| tp1_k13 | K13. Isolation and treatment of people who are infected with the COVID-19 virus are effective ways to reduce the spread of the virus. | 1, True \| 0, False \| 2, Don't know |
| tp1_k14 | K14. People who have contact with someone infected with the COVID-19 virus should be immediately isolated in a proper place for 14 days. | 1, True \| 0, False \| 2, Don't know |
| tp1_a1 | A1. I am worried about getting infected with the COVID-19 virus. | 1 Agree  0 Disagree  2 Don't know |
| tp1_a2 | A2. I feel confident I can prevent myself and my family from becoming infected with the COVID-19 virus. | 1 Agree  0 Disagree  2 Don't know |
| tp1_a3 | A3. I know what actions to take to prevent myself and my family from becoming infected with the COVID-19 virus. | 1 Agree  0 Disagree  2 Don't know |
| tp1_a4 | A4. I support CDC imposed guidelines for those who are infected with the COVID-19 virus. | 1 Agree  0 Disagree  2 Don't know |
| tp1_a5 | A5. I support/would support city and state-imposed regulations to protect the public (e.g. business closures, mask wearing, park closures, beach closures, etc.) | 1 Agree  0 Disagree  2 Don't know |
| tp1_a6 | A6. I support postponing or canceling mass gatherings such as concerts, festivals, and sporting events. | 1 Agree  0 Disagree  2 Don't know |
| tp1_a7 | A7. I support closure of K-12 schools if any student, staff member, or teacher is found to have COVID-19. | 1 Agree  0 Disagree  2 Don't know |
| tp1_a8 | A8. If I were exposed to and could possibly be infected with the COVID-19 virus, I would be willing to quarantine myself at home for 2 weeks until I was sure I was not infected in order to prevent others from getting COVID-19 from me. | 1 Agree  0 Disagree  2 Don’t know |
| tp1_p1 | P1. In recent days, I am washing my hands with soap and water more often than normal. | 1, Yes\| 0, No |
| tp1_p2 | P2. In recent days, I am using more disinfectants, such as hand sanitizers and cloth wipes. | 1, Yes\| 0, No |
| tp1_p3 | P3. In recent days, I am avoiding shaking hands or other physical contact with others outside my home. | 1, Yes\| 0, No |
| tp1_p4 | P4. In recent days, I have adhered to social distancing guidelines, such as avoiding meetings of more than 10 people and keeping a distance of 6 feet apart. | 1, Yes\| 0, No |
| tp1_p5 | P5. In recent days, I have bought larger amounts of staple foods (flour, sugar, pasta, rice, canned food) than normal. | 1, Yes\| 0, No |
| tp1_pscm1 | 1. I worry about many different things | radio, Required   \| 1 \| Never \| \| --- \| --- \| \| 2 \| Occasionally \| \| 3 \| Half the Time \| \| 4 \| Often \| \| 5 \| Always \| |
| tp1_pscm2 | 2. I am irritable and have a short temper | radio, Required   \| 1 \| Never \| \| --- \| --- \| \| 2 \| Occasionally \| \| 3 \| Half the Time \| \| 4 \| Often \| \| 5 \| Always \| |
| tp1_pscm3 | 3. I have unexplained physical ailments (headaches, digestive problems, dizziness) | radio, Required   \| 1 \| Never \| \| --- \| --- \| \| 2 \| Occasionally \| \| 3 \| Half the Time \| \| 4 \| Often \| \| 5 \| Always \| |
| tp1_pscm4 | 4. I am having a hard time: sleeping, eating, interacting with others positively | radio, Required   \| 1 \| Never \| \| --- \| --- \| \| 2 \| Occasionally \| \| 3 \| Half the Time \| \| 4 \| Often \| \| 5 \| Always \| |
| tp1_pscm5 | 1. I listen to the experts and follow their advice. | radio, Required   \| 1 \| Strongly disagree \| \| --- \| --- \| \| 2 \| Disagree \| \| 3 \| Neither agree or disagree \| \| 4 \| Agree \| \| 5 \| Strongly agree \| |
| tp1_pscm6 | 2. I think carefully about what to do and stick to it. | radio, Required   \| 1 \| Strongly disagree \| \| --- \| --- \| \| 2 \| Disagree \| \| 3 \| Neither agree or disagree \| \| 4 \| Agree \| \| 5 \| Strongly agree \| |
| tp1_pscm7 | 3. I try not to do anything rash. | radio, Required   \| 1 \| Strongly disagree \| \| --- \| --- \| \| 2 \| Disagree \| \| 3 \| Neither agree or disagree \| \| 4 \| Agree \| \| 5 \| Strongly agree \| |
| tp1_pscm8 | 4. I focus on what to do next. | radio, Required   \| 1 \| Strongly disagree \| \| --- \| --- \| \| 2 \| Disagree \| \| 3 \| Neither agree or disagree \| \| 4 \| Agree \| \| 5 \| Strongly agree \| |
| tp1_pscm9 | 5. I focus on what I will do next. | radio, Required   \| 1 \| Strongly disagree \| \| --- \| --- \| \| 2 \| Disagree \| \| 3 \| Neither agree or disagree \| \| 4 \| Agree \| \| 5 \| Strongly agree \| |
| tp1_pscm10 | 6. I talk to others to learn more about the situation. | radio, Required   \| 1 \| Strongly disagree \| \| --- \| --- \| \| 2 \| Disagree \| \| 3 \| Neither agree or disagree \| \| 4 \| Agree \| \| 5 \| Strongly agree \| |
| tp1_pscm11 | 7. I know what to do and try to do everything with twice the effort. | radio, Required   \| 1 \| Strongly disagree \| \| --- \| --- \| \| 2 \| Disagree \| \| 3 \| Neither agree or disagree \| \| 4 \| Agree \| \| 5 \| Strongly agree \| |
| tp1_pscm12 | 8. I change things in my life to be able to cope better with it all. | radio, Required   \| 1 \| Strongly disagree \| \| --- \| --- \| \| 2 \| Disagree \| \| 3 \| Neither agree or disagree \| \| 4 \| Agree \| \| 5 \| Strongly agree \| |
| tp1_pscm13 | 9. I have repeatedly thought about it and try to understand it. | radio, Required   \| 1 \| Strongly disagree \| \| --- \| --- \| \| 2 \| Disagree \| \| 3 \| Neither agree or disagree \| \| 4 \| Agree \| \| 5 \| Strongly agree \| |
| tp1_pscm14 | 10. I have been thinking about what I usually do with other viral infections. | radio, Required   \| 1 \| Strongly disagree \| \| --- \| --- \| \| 2 \| Disagree \| \| 3 \| Neither agree or disagree \| \| 4 \| Agree \| \| 5 \| Strongly agree \| |
| tp1_pscm15 | 11. I talk to someone who knows about it. | radio, Required   \| 1 \| Strongly disagree \| \| --- \| --- \| \| 2 \| Disagree \| \| 3 \| Neither agree or disagree \| \| 4 \| Agree \| \| 5 \| Strongly agree \| |
| tp1_pscm16 | 12. I am doing something completely new that I would never have done in other circumstances. | radio, Required   \| 1 \| Strongly disagree \| \| --- \| --- \| \| 2 \| Disagree \| \| 3 \| Neither agree or disagree \| \| 4 \| Agree \| \| 5 \| Strongly agree \| |
| tp1_pscm17 | 13. I ask for advice from highly respected people and adhere to it. | radio, Required   \| 1 \| Strongly disagree \| \| --- \| --- \| \| 2 \| Disagree \| \| 3 \| Neither agree or disagree \| \| 4 \| Agree \| \| 5 \| Strongly agree \| |
| tp1_pscm18 | 14. I have seen something like this before. | radio, Required   \| 1 \| Strongly disagree \| \| --- \| --- \| \| 2 \| Disagree \| \| 3 \| Neither agree or disagree \| \| 4 \| Agree \| \| 5 \| Strongly agree \| |
| tp1_pscm19 | 1. It will emerge over time; there is nothing more to do but wait. | radio, Required   \| 1 \| Strongly disagree \| \| --- \| --- \| \| 2 \| Disagree \| \| 3 \| Neither agree or disagree \| \| 4 \| Agree \| \| 5 \| Strongly agree \| |
| tp1_pscm20 | 2. I turn to my work or other activities to distract myself. | radio, Required   \| 1 \| Strongly disagree \| \| --- \| --- \| \| 2 \| Disagree \| \| 3 \| Neither agree or disagree \| \| 4 \| Agree \| \| 5 \| Strongly agree \| |
| tp1_pscm21 | 3. I imagine how the whole thing could end. | radio, Required   \| 1 \| Strongly disagree \| \| --- \| --- \| \| 2 \| Disagree \| \| 3 \| Neither agree or disagree \| \| 4 \| Agree \| \| 5 \| Strongly agree \| |
| tp1_pscm22 | 4. I imagine things that improve my mood. | radio, Required   \| 1 \| Strongly disagree \| \| --- \| --- \| \| 2 \| Disagree \| \| 3 \| Neither agree or disagree \| \| 4 \| Agree \| \| 5 \| Strongly agree \| |
| tp1_pscm23 | 5. I submit to my fate; sometimes you are just unlucky. | radio, Required   \| 1 \| Strongly disagree \| \| --- \| --- \| \| 2 \| Disagree \| \| 3 \| Neither agree or disagree \| \| 4 \| Agree \| \| 5 \| Strongly agree \| |
| tp1_pscm24 | 6. I tell myself things that make it easier for me. | radio, Required   \| 1 \| Strongly disagree \| \| --- \| --- \| \| 2 \| Disagree \| \| 3 \| Neither agree or disagree \| \| 4 \| Agree \| \| 5 \| Strongly agree \| |
| tp1_pscm25 | 7. I do things that are probably of no use, but I feel like at least I am doing something. | radio, Required   \| 1 \| Strongly disagree \| \| --- \| --- \| \| 2 \| Disagree \| \| 3 \| Neither agree or disagree \| \| 4 \| Agree \| \| 5 \| Strongly agree \| |
| tp1_pscm26 | 8. I wish I could change my worries and feelings. | radio, Required   \| 1 \| Strongly disagree \| \| --- \| --- \| \| 2 \| Disagree \| \| 3 \| Neither agree or disagree \| \| 4 \| Agree \| \| 5 \| Strongly agree \| |
| tp1_pscm27 | 9. I hope for a miracle. | radio, Required   \| 1 \| Strongly disagree \| \| --- \| --- \| \| 2 \| Disagree \| \| 3 \| Neither agree or disagree \| \| 4 \| Agree \| \| 5 \| Strongly agree \| |
| tp1_pscm28 | 10. I try to make myself feel better by eating, drinking, smoking or taking medication. | radio, Required   \| 1 \| Strongly disagree \| \| --- \| --- \| \| 2 \| Disagree \| \| 3 \| Neither agree or disagree \| \| 4 \| Agree \| \| 5 \| Strongly agree \| |
| tp1_pscm29 | 11. I take refuge in daydreams and imagine times when it was better than today. | radio, Required   \| 1 \| Strongly disagree \| \| --- \| --- \| \| 2 \| Disagree \| \| 3 \| Neither agree or disagree \| \| 4 \| Agree \| \| 5 \| Strongly agree \| |
| tp1_pscm30 | 12. I try to leave the whole thing behind and want to rest or go on vacation. | radio, Required   \| 1 \| Strongly disagree \| \| --- \| --- \| \| 2 \| Disagree \| \| 3 \| Neither agree or disagree \| \| 4 \| Agree \| \| 5 \| Strongly agree \| |
| tp1_pscm31 | 13. I refuse to believe what is happening. | radio, Required   \| 1 \| Strongly disagree \| \| --- \| --- \| \| 2 \| Disagree \| \| 3 \| Neither agree or disagree \| \| 4 \| Agree \| \| 5 \| Strongly agree \| |
| tp1_work | Is any adult in your child's home currently employed? | yesno, Required   \| 1 \| Yes \| \| --- \| --- \| \| 0 \| No \| |
| tp1_work_status | If employed: | radio, Required   \| 1 \| Full time \| \| --- \| --- \| \| 2 \| Part-time \| |
| tp1_work_med | Does anyone in your child's home work or volunteer in a hospital or other medical setting? | yesno, Required   \| 1 \| Yes \| \| --- \| --- \| \| 0 \| No \| |
| tp1_lostincome | Has a primary wage earner within your child's household lost their job or regular income source due to COVID-19? | yesno, Required   \| 1 \| Yes \| \| --- \| --- \| \| 0 \| No \| |
| tp1_flu_vacc | Q05. Has your child ever received a flu vaccine? | yesno, Required   \| 1 \| Yes \| \| --- \| --- \| \| 0 \| No \| |
| tp1_flu_vacc_19  Show the field ONLY if:  [tp1_flu_vacc] = '1' | Q06. Did your child receive the flu vaccine in the Fall of 2019 or Winter/Spring of 2020? | yesno, Required   \| 1 \| Yes \| \| --- \| --- \| \| 0 \| No \| |
| tp1_flu_vacc_method  Show the field ONLY if:  [tp1_flu_vacc_19] = '1' | If yes, was it injection or nasal? | radio, Required   \| 1 \| Injection \| \| --- \| --- \| \| 2 \| Nasal \| |
| **The following questions are answered by the children or by the parents who facilitate the questions for the children. The instructions that appear are as follows:**  **For children under 8 In consultation with your child, please have him/her answer the following questions:For children aged 8-12: Please allow your child to read and answer the following questions his/herself:** | | |
| tp1_cscm1 | 1. I feel hopeless and sad (about the virus) | radio, Required   \| 1 \| Never \| \| --- \| --- \| \| 2 \| A little \| \| 3 \| Sometimes \| \| 4 \| A lot \| \| 5 \| Always \| |
| tp1_cscm2 | 2. I have trouble eating or sleeping | radio, Required   \| 1 \| Never \| \| --- \| --- \| \| 2 \| A little \| \| 3 \| Sometimes \| \| 4 \| A lot \| \| 5 \| Always \| |
| tp1_cscm3 | 3. I find myself crying a lot | radio, Required   \| 1 \| Never \| \| --- \| --- \| \| 2 \| A little \| \| 3 \| Sometimes \| \| 4 \| A lot \| \| 5 \| Always \| |
| tp1_cscm4 | 4. I feel worried or nervous (about the virus) | radio, Required   \| 1 \| Never \| \| --- \| --- \| \| 2 \| A little \| \| 3 \| Sometimes \| \| 4 \| A lot \| \| 5 \| Always \| |
| tp1_cscm5 | 5. It is hard to stop my thoughts (about the virus) | radio, Required   \| 1 \| Never \| \| --- \| --- \| \| 2 \| A little \| \| 3 \| Sometimes \| \| 4 \| A lot \| \| 5 \| Always \| |
| tp1_cscm6 | 6. I cannot stop worrying (about the virus) | radio, Required   \| 1 \| Never \| \| --- \| --- \| \| 2 \| A little \| \| 3 \| Sometimes \| \| 4 \| A lot \| \| 5 \| Always \| |
| tp1_cscm7 | 7. I am very scared of getting dirty | radio, Required   \| 1 \| Never \| \| --- \| --- \| \| 2 \| A little \| \| 3 \| Sometimes \| \| 4 \| A lot \| \| 5 \| Always \| |
| tp1_cscm8 | 8. I have to wash my hands, over and over to feel better | radio, Required   \| 1 \| Never \| \| --- \| --- \| \| 2 \| A little \| \| 3 \| Sometimes \| \| 4 \| A lot \| \| 5 \| Always \| |
| tp1_cscm9 | 9. I have a stomachache/headache | radio, Required   \| 1 \| Never \| \| --- \| --- \| \| 2 \| A little \| \| 3 \| Sometimes \| \| 4 \| A lot \| \| 5 \| Always \| |
| tp1_cscm10 | I0. It's hard for me to think a long time | radio, Required   \| 1 \| Never \| \| --- \| --- \| \| 2 \| A little \| \| 3 \| Sometimes \| \| 4 \| A lot \| \| 5 \| Always \| |
| tp1_cscm11 | 1. What do you feel when you think of the virus? | text, Required |
| tp1_cscm12 | 2. How can you help yourself feel better? | text, Required |
| tp1_cscm13 | 3. How would you help a friend if they were feeling scared about the virus? | text, Required |
| tp1_cscm14 | 4. How is school helping you with the virus? | text, Required |
| tp1_cscm15 | 5. What else could help you with your feelings about the virus? | text, Required |
| tp1_cscm16 | 6. Would you like to read about ways to feel better about the virus? | text, Required |
| **The following variables are asked to children 13 years of age or older.** | | |
| tp1_13cscm2 | 1. Sadness, feeling down, low mood, feeling fatigued | radio, Required   \| 1 \| Never \| \| --- \| --- \| \| 2 \| Occasionally \| \| 3 \| Half the Time \| \| 4 \| Often \| \| 5 \| Always \| |
| tp1_13cscm3 | 2. Feelings of hopelessness, worthlessness, emptiness, or not being a good person | radio, Required   \| 1 \| Never \| \| --- \| --- \| \| 2 \| Occasionally \| \| 3 \| Half the Time \| \| 4 \| Often \| \| 5 \| Always \| |
| tp1_13cscm4 | 3. Decreased pleasure from things that used to be fun, feeling that life is not much fun | radio, Required   \| 1 \| Never \| \| --- \| --- \| \| 2 \| Occasionally \| \| 3 \| Half the Time \| \| 4 \| Often \| \| 5 \| Always \| |
| tp1_13cscm5 | 4. Feeling worried, nervous, panicky, tense, keyed-up | radio, Required   \| 1 \| Never \| \| --- \| --- \| \| 2 \| Occasionally \| \| 3 \| Half the Time \| \| 4 \| Often \| \| 5 \| Always \| |
| tp1_13cscm6 | 5.Not being able to stop worrying or controlling your worry | radio, Required   \| 1 \| Never \| \| --- \| --- \| \| 2 \| Occasionally \| \| 3 \| Half the Time \| \| 4 \| Often \| \| 5 \| Always \| |
| tp1_13cscm7 | 6. Being easily annoyed or irritable, feelings of dread like something awful might happen | radio, Required   \| 1 \| Never \| \| --- \| --- \| \| 2 \| Occasionally \| \| 3 \| Half the Time \| \| 4 \| Often \| \| 5 \| Always \| |
| tp1_13cscm8 | 7. Constant thoughts about avoiding germs | radio, Required   \| 1 \| Never \| \| --- \| --- \| \| 2 \| Occasionally \| \| 3 \| Half the Time \| \| 4 \| Often \| \| 5 \| Always \| |
| tp1_13cscm9 | 8. Fixation with washing your hands throughout the day | radio, Required   \| 1 \| Never \| \| --- \| --- \| \| 2 \| Occasionally \| \| 3 \| Half the Time \| \| 4 \| Often \| \| 5 \| Always \| |
| tp1_13cscm10 | 9. Sudden moments of fear or terror because you couldn't get rid of the germs | radio, Required   \| 1 \| Never \| \| --- \| --- \| \| 2 \| Occasionally \| \| 3 \| Half the Time \| \| 4 \| Often \| \| 5 \| Always \| |
| tp1_13cscm11 | 10. Felt a racing heart, shaky sweaty, or had trouble breathing | radio, Required   \| 1 \| Never \| \| --- \| --- \| \| 2 \| Occasionally \| \| 3 \| Half the Time \| \| 4 \| Often \| \| 5 \| Always \| |
| tp1_13cscm18 | Please share your thoughts about the following questions | descriptive |
| tp1_13cscm12 | 1. If you feel stressed or scared about the coronavirus, what seems to help you feel better? | text, Required |
| tp1_13cscm13 | 2. How do you help yourself feel calm when you hear about how the virus is spreading? | text, Required |
| tp1_13cscm14 | 3. How do you help your friends that are feeling scared about the coronavirus? | text, Required |
| tp1_13cscm15 | 4. What parts of school are helping you feel supported about your feelings about the coronavirus? | text, Required |
| tp1_13cscm16 | 5. How does continuing to see your peers and teachers help you during this pandemic? | text, Required |
| tp1_13cscm17 | 6. What types of handouts or readings would you be interested in about the coronavirus? | text, Required |

### TP2 Questionnaire

| Variable name | Question asked participants | Possible answers/Code |
| --- | --- | --- |
| tp2_reported_income | Q3) Total Family Annual Income (use 2019 estimate) $ | Numeric value |
| **Most KAP remain the same as for tp1 (only difference being the timepoint, and a re-coding of responses for attitudes (from 3 point to 5-point Likert Scale). Furthermore, additional questions where included in TP2 and TP3. Below are the additional questions added, and the ones that were recoded (attitude). See tp1 table for the questions that remained throughout the three timepoints.** | | |
| tp2_k15 | K15. People with a strong immune system will not get infected with COVID-19. | radio, Required   \| 1 \| True \| \| --- \| --- \| \| 0 \| False \| \| 2 \| Don't know \| |
| tp2_k16 | K16. To prevent the infection by COVID-19, individuals should avoid going to crowded places such as restaurants, bars, concerts, etc. | radio, Required   \| 1 \| True \| \| --- \| --- \| \| 0 \| False \| \| 2 \| Don't know \| |
| tp2_a1 | A1. I am worried about getting infected with the COVID-19 virus. | radio, Required   \| 1 \| Strongly disagree \| \| --- \| --- \| \| 2 \| Disagree \| \| 3 \| Neither agree nor disagree \| \| 4 \| Agree \| \| 5 \| Strongly agree \| |
| tp2_a2 | A2. I feel confident I can prevent myself and my family from becoming infected with the COVID-19 virus. | radio, Required   \| 1 \| Strongly disagree \| \| --- \| --- \| \| 2 \| Disagree \| \| 3 \| Neither agree nor disagree \| \| 4 \| Agree \| \| 5 \| Strongly agree \| |
| tp2_a3 | A3. I know what actions to take to prevent myself and my family from becoming infected with the COVID-19 virus. | radio, Required   \| 1 \| Strongly disagree \| \| --- \| --- \| \| 2 \| Disagree \| \| 3 \| Neither agree nor disagree \| \| 4 \| Agree \| \| 5 \| Strongly agree \| |
| tp2_a4 | A4. I support CDC imposed guidelines for those who are infected with the COVID-19 virus. | radio, Required   \| 1 \| Strongly disagree \| \| --- \| --- \| \| 2 \| Disagree \| \| 3 \| Neither agree nor disagree \| \| 4 \| Agree \| \| 5 \| Strongly agree \| |
| tp2_a5 | A5. I support/would support city and state-imposed regulations to protect the public (e.g. business closures, mask wearing, park closures, beach closures, etc.) | radio, Required   \| 1 \| Strongly disagree \| \| --- \| --- \| \| 2 \| Disagree \| \| 3 \| Neither agree nor disagree \| \| 4 \| Agree \| \| 5 \| Strongly agree \| |
| tp2_a6 | A6. I support postponing or canceling mass gatherings such as concerts, festivals, and sporting events. | radio, Required   \| 1 \| Strongly disagree \| \| --- \| --- \| \| 2 \| Disagree \| \| 3 \| Neither agree nor disagree \| \| 4 \| Agree \| \| 5 \| Strongly agree \| |
| tp2_a7 | A7. I support closure of K-12 schools if any student, staff member, or teacher is found to have COVID-19. | radio, Required   \| 1 \| Strongly disagree \| \| --- \| --- \| \| 2 \| Disagree \| \| 3 \| Neither agree nor disagree \| \| 4 \| Agree \| \| 5 \| Strongly agree \| |
| tp2_a8 | A8. If I were exposed to and could possibly be infected with the COVID-19 virus, I would be willing to quarantine myself at home for 2 weeks until I was sure I was not infected in order to prevent others from getting COVID-19 from me. | radio, Required   \| 1 \| Strongly disagree \| \| --- \| --- \| \| 2 \| Disagree \| \| 3 \| Neither agree nor disagree \| \| 4 \| Agree \| \| 5 \| Strongly agree \| |
| tp2_a9 | A9. I support K-12 schools re-opening in person instruction this fall. | radio, Required   \| 1 \| Strongly disagree \| \| --- \| --- \| \| 2 \| Disagree \| \| 3 \| Neither agree nor disagree \| \| 4 \| Agree \| \| 5 \| Strongly agree \| |
| tp2_a10 | A10. I am comfortable going out in public as long as I am in open spaces. | radio, Required   \| 1 \| Strongly disagree \| \| --- \| --- \| \| 2 \| Disagree \| \| 3 \| Neither agree nor disagree \| \| 4 \| Agree \| \| 5 \| Strongly agree \| |
| tp2_a11 | A11. I am comfortable going into essential public spaces as necessary, such as grocery stores, pharmacies, etc. | radio, Required   \| 1 \| Strongly disagree \| \| --- \| --- \| \| 2 \| Disagree \| \| 3 \| Neither agree nor disagree \| \| 4 \| Agree \| \| 5 \| Strongly agree \| |
| tp2_a12 | A12. I am comfortable going into public retail spaces, such as department stores, restaurants, etc. | radio, Required   \| 1 \| Strongly disagree \| \| --- \| --- \| \| 2 \| Disagree \| \| 3 \| Neither agree nor disagree \| \| 4 \| Agree \| \| 5 \| Strongly agree \| |
| tp2_a13 | A13. I think that the US government is handling the COVID-19 health crisis well. | radio, Required   \| 1 \| Strongly disagree \| \| --- \| --- \| \| 2 \| Disagree \| \| 3 \| Neither agree nor disagree \| \| 4 \| Agree \| \| 5 \| Strongly agree \| |
| tp2_a14 | A14. I support mask mandates in closed-in areas where physical distancing (6 ft) is not feasible (stores, offices, schools, etc.) | radio, Required   \| 1 \| Strongly disagree \| \| --- \| --- \| \| 2 \| Disagree \| \| 3 \| Neither agree nor disagree \| \| 4 \| Agree \| \| 5 \| Strongly agree \| |
| tp2_p6 | P6. I am avoiding going into any crowded place. | yesno, Required   \| 1 \| Yes \| \| --- \| --- \| \| 0 \| No \| |
| tp2_p7 | P7. I wear a mask when leaving home and entering an indoor area with other people. | yesno, Required   \| 1 \| Yes \| \| --- \| --- \| \| 0 \| No \| |
| tp2_p8 | P8. In the past 7 days I have stayed at home or worked from home rather than going into work. | yesno, Required   \| 1 \| Yes \| \| --- \| --- \| \| 0 \| No \| |
| **tp2_cscm1 to tp2_cscm31 remain the same as for tp1 (only difference being the timepoint). See tp1 table for questions asked to participants and the possible answers/code.** | | |
| tp2_vax_att1 | V1. Do you plan on having your child(ren) vaccinated against COVID-19 (coronavirus/SARS-CoV-2) when a vaccine becomes available? | radio, Required   \| 1 \| Yes \| \| --- \| --- \| \| 2 \| Unsure/Haven't Decided Yet \| \| 0 \| No \| |
| tp2_vax_att2 | V2. How concerned are you that the coronavirus vaccine may not be safe for your child(ren)? | radio, Required   \| 1 \| Very Concerned \| \| --- \| --- \| \| 2 \| Somewhat Concerned \| \| 3 \| Not Sure \| \| 4 \| Not Too Concerned \| \| 5 \| Not at All Concerned \| |
| tp2_vax_att3 | V3. How concerned are you that your child(ren) may have a serious side effect from the coronavirus vaccine? | radio, Required   \| 1 \| Very Concerned \| \| --- \| --- \| \| 2 \| Somewhat Concerned \| \| 3 \| Not Sure \| \| 4 \| Not Too Concerned \| \| 5 \| Not at All Concerned \| |
| tp2_vax_att4 | V4. The information I receive about the coronavirus vaccine from the news/media is reliable and trustworthy. | radio, Required   \| 1 \| Strongly Disagree \| \| --- \| --- \| \| 2 \| Disagree \| \| 3 \| Not Sure \| \| 4 \| Agree \| \| 5 \| Strongly Agree \| |
| tp2_vax_att5 | V5. I am able to openly discuss my concerns about the coronavirus vaccine with my child(ren)'s doctor. | radio, Required   \| 1 \| Strongly Disagree \| \| --- \| --- \| \| 2 \| Disagree \| \| 3 \| Not Sure \| \| 4 \| Agree \| \| 5 \| Strongly Agree \| |
| tp2_vax_att6 | V6. The coronavirus vaccine will be effective at preventing COVID-19. | radio, Required   \| 1 \| Strongly Disagree \| \| --- \| --- \| \| 2 \| Disagree \| \| 3 \| Not Sure \| \| 4 \| Agree \| \| 5 \| Strongly Agree \| |
| tp2_vax_att7 | V7. Having my child(ren) vaccinated against the coronavirus is important for the health of others in my community. | radio, Required   \| 1 \| Strongly Disagree \| \| --- \| --- \| \| 2 \| Disagree \| \| 3 \| Not Sure \| \| 4 \| Agree \| \| 5 \| Strongly Agree \| |
| tp2_cdc1 | 1. Would you say in general that your health is: | radio, Required   \| 1 \| Excellent \| \| --- \| --- \| \| 2 \| Very good \| \| 3 \| Good \| \| 4 \| Fair \| \| 5 \| Poor \| |
| tp2_cdc2 | 2. Now thinking about your physical health, which includes physical illness and injury, for how many days during the past 30 days was your physical health not good?  *If none, enter '0' and continue.* | text (number, Min: 0, Max: 30), Required |
| tp2_cdc3 | 3. Now thinking about your mental health, which includes stress, depression, and problems with emotions, for how many days during the past 30 days was your mental health not good?  *If none, enter '0' and continue.* | text (number, Min: 0, Max: 30), Required |
| tp2_cdc4 | 4. During the past 30 days, for about how many days did poor physical or mental health keep you from doing your usual activities, such as self-care, work, or recreation?  *If none, enter '0' and continue.* | text (number, Min: 0, Max: 30), Required |
| tp2_optimism1 | 1. In uncertain times, I usually expect the best. | radio, Required   \| 1 \| I agree a lot \| \| --- \| --- \| \| 2 \| I agree a little \| \| 3 \| I neither agree nor disagree \| \| 4 \| I disagree a little \| \| 5 \| I disagree a lot \| |
| tp2_optimism2 | 2. It's easy for me to relax. | radio, Required   \| 1 \| I agree a lot \| \| --- \| --- \| \| 2 \| I agree a little \| \| 3 \| I neither agree nor disagree \| \| 4 \| I disagree a little \| \| 5 \| I disagree a lot \| |
| tp2_optimism3 | 3. If something can go wrong for me, it will. | radio, Required   \| 1 \| I agree a lot \| \| --- \| --- \| \| 2 \| I agree a little \| \| 3 \| I neither agree nor disagree \| \| 4 \| I disagree a little \| \| 5 \| I disagree a lot \| |
| tp2_optimism4 | 4. I'm always optimistic about my future. | radio, Required   \| 1 \| I agree a lot \| \| --- \| --- \| \| 2 \| I agree a little \| \| 3 \| I neither agree nor disagree \| \| 4 \| I disagree a little \| \| 5 \| I disagree a lot \| |
| tp2_optimism5 | 5. I enjoy my friends a lot. | radio, Required   \| 1 \| I agree a lot \| \| --- \| --- \| \| 2 \| I agree a little \| \| 3 \| I neither agree nor disagree \| \| 4 \| I disagree a little \| \| 5 \| I disagree a lot \| |
| tp2_optimism6 | 6. It's important for me to keep busy. | radio, Required   \| 1 \| I agree a lot \| \| --- \| --- \| \| 2 \| I agree a little \| \| 3 \| I neither agree nor disagree \| \| 4 \| I disagree a little \| \| 5 \| I disagree a lot \| |
| tp2_optimism7 | 7. I hardly ever expect things to go my way. | radio, Required   \| 1 \| I agree a lot \| \| --- \| --- \| \| 2 \| I agree a little \| \| 3 \| I neither agree nor disagree \| \| 4 \| I disagree a little \| \| 5 \| I disagree a lot \| |
| tp2_optimism8 | 8. I don't get upset too easily. | radio, Required   \| 1 \| I agree a lot \| \| --- \| --- \| \| 2 \| I agree a little \| \| 3 \| I neither agree nor disagree \| \| 4 \| I disagree a little \| \| 5 \| I disagree a lot \| |
| tp2_optimism9 | 9. I rarely count on good things happening to me. | radio, Required   \| 1 \| I agree a lot \| \| --- \| --- \| \| 2 \| I agree a little \| \| 3 \| I neither agree nor disagree \| \| 4 \| I disagree a little \| \| 5 \| I disagree a lot \| |
| tp2_optimism10 | 10. Overall, I expect more good things to happen to me than bad. | radio, Required   \| 1 \| I agree a lot \| \| --- \| --- \| \| 2 \| I agree a little \| \| 3 \| I neither agree nor disagree \| \| 4 \| I disagree a little \| \| 5 \| I disagree a lot \| |
| tp2_h1text | H1. How easy or difficult would you say it is to: |  |
| tp2_h1a | 1. Find the information you need related to COVID-19? | radio (Matrix), Required   \| 1 \| 1 Very difficult \| \| --- \| --- \| \| 2 \| 2 \| \| 3 \| 3 \| \| 4 \| 4 \| \| 5 \| 5 \| \| 6 \| 6 \| \| 7 \| 7 Very easy \| |
| tp2_h1b | 2. Understand information about what to do if you think you have COVID-19? | radio (Matrix), Required   \| 1 \| 1 Very difficult \| \| --- \| --- \| \| 2 \| 2 \| \| 3 \| 3 \| \| 4 \| 4 \| \| 5 \| 5 \| \| 6 \| 6 \| \| 7 \| 7 Very easy \| |
| tp2_h1c | 3. Judge if the information about COVID-19 in the media is reliable | radio (Matrix), Required   \| 1 \| 1 Very difficult \| \| --- \| --- \| \| 2 \| 2 \| \| 3 \| 3 \| \| 4 \| 4 \| \| 5 \| 5 \| \| 6 \| 6 \| \| 7 \| 7 Very easy \| |
| tp2_h1d | 4. Understand restrictions and recommendations of authorities regarding COVID-19 | radio (Matrix), Required   \| 1 \| 1 Very difficult \| \| --- \| --- \| \| 2 \| 2 \| \| 3 \| 3 \| \| 4 \| 4 \| \| 5 \| 5 \| \| 6 \| 6 \| \| 7 \| 7 Very easy \| |
| tp2_h1e | 5. Follow the recommendations on how to protect yourself from COVID-19 | radio (Matrix), Required   \| 1 \| 1 Very difficult \| \| --- \| --- \| \| 2 \| 2 \| \| 3 \| 3 \| \| 4 \| 4 \| \| 5 \| 5 \| \| 6 \| 6 \| \| 7 \| 7 Very easy \| |
| tp2_h1f | 6. Understand recommendations about when to stay at home from work/school, and when not to? | radio (Matrix), Required   \| 1 \| 1 Very difficult \| \| --- \| --- \| \| 2 \| 2 \| \| 3 \| 3 \| \| 4 \| 4 \| \| 5 \| 5 \| \| 6 \| 6 \| \| 7 \| 7 Very easy \| |
| tp2_h1g | 7. Follow recommendations about when to stay at home from work/school, and when not to? | radio (Matrix), Required   \| 1 \| 1 Very difficult \| \| --- \| --- \| \| 2 \| 2 \| \| 3 \| 3 \| \| 4 \| 4 \| \| 5 \| 5 \| \| 6 \| 6 \| \| 7 \| 7 Very easy \| |
| tp2_h1h | 8. Understand recommendations about when to engage in social activities, and when not to? | radio (Matrix), Required   \| 1 \| 1 Very difficult \| \| --- \| --- \| \| 2 \| 2 \| \| 3 \| 3 \| \| 4 \| 4 \| \| 5 \| 5 \| \| 6 \| 6 \| \| 7 \| 7 Very easy \| |
| tp2_h1i | 9. Follow recommendations about when to engage in social activities, and when not to? | radio (Matrix), Required   \| 1 \| 1 Very difficult \| \| --- \| --- \| \| 2 \| 2 \| \| 3 \| 3 \| \| 4 \| 4 \| \| 5 \| 5 \| \| 6 \| 6 \| \| 7 \| 7 Very easy \| |
| tp2_h1j | 10. Obtain a COVID-19 Test | radio (Matrix), Required   \| 1 \| 1 Very difficult \| \| --- \| --- \| \| 2 \| 2 \| \| 3 \| 3 \| \| 4 \| 4 \| \| 5 \| 5 \| \| 6 \| 6 \| \| 7 \| 7 Very easy \| |
| tp2_h2text | H2. COVID-19 is... | descriptive |
| tp2_h2a | a) Close to me - Far away from me | radio (Matrix), Required   \| 1 \| 1 \| \| --- \| --- \| \| 2 \| 2 \| \| 3 \| 3 \| \| 4 \| 4 \| \| 5 \| 5 \| \| 6 \| 6 \| \| 7 \| 7 \| |
| tp2_h2b | b) Spreading slowly - Spreading fast | radio (Matrix), Required   \| 1 \| 1 \| \| --- \| --- \| \| 2 \| 2 \| \| 3 \| 3 \| \| 4 \| 4 \| \| 5 \| 5 \| \| 6 \| 6 \| \| 7 \| 7 \| |
| tp2_h2c | c) Something I think about all the time - Something I almost never think about | radio (Matrix), Required   \| 1 \| 1 \| \| --- \| --- \| \| 2 \| 2 \| \| 3 \| 3 \| \| 4 \| 4 \| \| 5 \| 5 \| \| 6 \| 6 \| \| 7 \| 7 \| |
| tp2_h2d | d) Scary - Not scary | radio (Matrix), Required   \| 1 \| 1 \| \| --- \| --- \| \| 2 \| 2 \| \| 3 \| 3 \| \| 4 \| 4 \| \| 5 \| 5 \| \| 6 \| 6 \| \| 7 \| 7 \| |
| tp2_h2e |  |  |
| tp2_h3text | H3. How much do you trust information about COVID-19 from the following sources? | descriptive |
| tp2_h3a | a) Television | radio (Matrix), Required   \| 1 \| 1 Very little trust \| \| --- \| --- \| \| 2 \| 2 \| \| 3 \| 3 \| \| 4 \| 4 \| \| 5 \| 5 \| \| 6 \| 6 \| \| 7 \| 7 A great deal of trust \| |
| tp2_h3b | b) Newspapers | radio (Matrix), Required   \| 1 \| 1 Very little trust \| \| --- \| --- \| \| 2 \| 2 \| \| 3 \| 3 \| \| 4 \| 4 \| \| 5 \| 5 \| \| 6 \| 6 \| \| 7 \| 7 A great deal of trust \| |
| tp2_h3c | c) Health workers | radio (Matrix), Required   \| 1 \| 1 Very little trust \| \| --- \| --- \| \| 2 \| 2 \| \| 3 \| 3 \| \| 4 \| 4 \| \| 5 \| 5 \| \| 6 \| 6 \| \| 7 \| 7 A great deal of trust \| |
| tp2_h3d | d) Social media | radio (Matrix), Required   \| 1 \| 1 Very little trust \| \| --- \| --- \| \| 2 \| 2 \| \| 3 \| 3 \| \| 4 \| 4 \| \| 5 \| 5 \| \| 6 \| 6 \| \| 7 \| 7 A great deal of trust \| |
| tp2_h3e | e) Radio | radio (Matrix), Required   \| 1 \| 1 Very little trust \| \| --- \| --- \| \| 2 \| 2 \| \| 3 \| 3 \| \| 4 \| 4 \| \| 5 \| 5 \| \| 6 \| 6 \| \| 7 \| 7 A great deal of trust \| |
| tp2_h3f | f) UF Health | radio (Matrix), Required   \| 1 \| 1 Very little trust \| \| --- \| --- \| \| 2 \| 2 \| \| 3 \| 3 \| \| 4 \| 4 \| \| 5 \| 5 \| \| 6 \| 6 \| \| 7 \| 7 A great deal of trust \| |
| tp2_h3g | g) Florida Department of Health | radio (Matrix), Required   \| 1 \| 1 Very little trust \| \| --- \| --- \| \| 2 \| 2 \| \| 3 \| 3 \| \| 4 \| 4 \| \| 5 \| 5 \| \| 6 \| 6 \| \| 7 \| 7 A great deal of trust \| |
| tp2_h3h | h) Centers for Disease Control and Prevention | radio (Matrix), Required   \| 1 \| 1 Very little trust \| \| --- \| --- \| \| 2 \| 2 \| \| 3 \| 3 \| \| 4 \| 4 \| \| 5 \| 5 \| \| 6 \| 6 \| \| 7 \| 7 A great deal of trust \| |
| tp2_h3i | i) Celebrities and social media influencers | radio (Matrix), Required   \| 1 \| 1 Very little trust \| \| --- \| --- \| \| 2 \| 2 \| \| 3 \| 3 \| \| 4 \| 4 \| \| 5 \| 5 \| \| 6 \| 6 \| \| 7 \| 7 A great deal of trust \| |
| tp2_h3j | j) World Health Organization (WHO) | radio (Matrix), Required   \| 1 \| 1 Very little trust \| \| --- \| --- \| \| 2 \| 2 \| \| 3 \| 3 \| \| 4 \| 4 \| \| 5 \| 5 \| \| 6 \| 6 \| \| 7 \| 7 A great deal of trust \| |
| tp2_h3k | k) COVID-19 Hotlines | radio (Matrix), Required   \| 1 \| 1 Very little trust \| \| --- \| --- \| \| 2 \| 2 \| \| 3 \| 3 \| \| 4 \| 4 \| \| 5 \| 5 \| \| 6 \| 6 \| \| 7 \| 7 A great deal of trust \| |
| tp2_h3l | l) US Government's National COVID-19 information website | radio (Matrix), Required   \| 1 \| 1 Very little trust \| \| --- \| --- \| \| 2 \| 2 \| \| 3 \| 3 \| \| 4 \| 4 \| \| 5 \| 5 \| \| 6 \| 6 \| \| 7 \| 7 A great deal of trust \| |
| tp2_h4text | H4. How often do you use the following sources for information about COVID-19? | descriptive |
| tp2_h4a | a) Television | radio (Matrix), Required   \| 1 \| 1 Never \| \| --- \| --- \| \| 2 \| 2 \| \| 3 \| 3 \| \| 4 \| 4 \| \| 5 \| 5 \| \| 6 \| 6 \| \| 7 \| 7 Very often \| |
| tp2_h4b | b) Newspapers | radio (Matrix), Required   \| 1 \| 1 Never \| \| --- \| --- \| \| 2 \| 2 \| \| 3 \| 3 \| \| 4 \| 4 \| \| 5 \| 5 \| \| 6 \| 6 \| \| 7 \| 7 Very often \| |
| tp2_h4c | c) Health workers | radio (Matrix), Required   \| 1 \| 1 Never \| \| --- \| --- \| \| 2 \| 2 \| \| 3 \| 3 \| \| 4 \| 4 \| \| 5 \| 5 \| \| 6 \| 6 \| \| 7 \| 7 Very often \| |
| tp2_h4d | d) Social media | radio (Matrix), Required   \| 1 \| 1 Never \| \| --- \| --- \| \| 2 \| 2 \| \| 3 \| 3 \| \| 4 \| 4 \| \| 5 \| 5 \| \| 6 \| 6 \| \| 7 \| 7 Very often \| |
| tp2_h4e | e) Radio stations | radio (Matrix), Required   \| 1 \| 1 Never \| \| --- \| --- \| \| 2 \| 2 \| \| 3 \| 3 \| \| 4 \| 4 \| \| 5 \| 5 \| \| 6 \| 6 \| \| 7 \| 7 Very often \| |
| tp2_h4f | f) UF Health | radio (Matrix), Required   \| 1 \| 1 Never \| \| --- \| --- \| \| 2 \| 2 \| \| 3 \| 3 \| \| 4 \| 4 \| \| 5 \| 5 \| \| 6 \| 6 \| \| 7 \| 7 Very often \| |
| tp2_h4g | g) Florida Department of Health | radio (Matrix), Required   \| 1 \| 1 Never \| \| --- \| --- \| \| 2 \| 2 \| \| 3 \| 3 \| \| 4 \| 4 \| \| 5 \| 5 \| \| 6 \| 6 \| \| 7 \| 7 Very often \| |
| tp2_h4h | h) Centers for Disease Control and Prevention (CDC) | radio (Matrix), Required   \| 1 \| 1 Never \| \| --- \| --- \| \| 2 \| 2 \| \| 3 \| 3 \| \| 4 \| 4 \| \| 5 \| 5 \| \| 6 \| 6 \| \| 7 \| 7 Very often \| |
| tp2_h4i | i) Celebrities and social media influencers | radio (Matrix), Required   \| 1 \| 1 Never \| \| --- \| --- \| \| 2 \| 2 \| \| 3 \| 3 \| \| 4 \| 4 \| \| 5 \| 5 \| \| 6 \| 6 \| \| 7 \| 7 Very often \| |
| tp2_h4j | j) World Health Organization (WHO) | radio (Matrix), Required   \| 1 \| 1 Never \| \| --- \| --- \| \| 2 \| 2 \| \| 3 \| 3 \| \| 4 \| 4 \| \| 5 \| 5 \| \| 6 \| 6 \| \| 7 \| 7 Very often \| |
| tp2_h4k | k) COVID-19 Hotlines | radio (Matrix), Required   \| 1 \| 1 Never \| \| --- \| --- \| \| 2 \| 2 \| \| 3 \| 3 \| \| 4 \| 4 \| \| 5 \| 5 \| \| 6 \| 6 \| \| 7 \| 7 Very often \| |
| tp2_h4l | l) US Government's National COVID-19 information website | radio (Matrix), Required   \| 1 \| 1 Never \| \| --- \| --- \| \| 2 \| 2 \| \| 3 \| 3 \| \| 4 \| 4 \| \| 5 \| 5 \| \| 6 \| 6 \| \| 7 \| 7 Very often \| |
| tp2_h5text | H5. How much confidence do you have that the following can handle the COVID-19 challenge well? | descriptive |
| tp2_h5a | a) Your family doctors | radio (Matrix), Required   \| 1 \| 1 Very low confidence \| \| --- \| --- \| \| 2 \| 2 \| \| 3 \| 3 \| \| 4 \| 4 \| \| 5 \| 5 \| \| 6 \| 6 \| \| 7 \| 7 Very high confidence \| \| 8 \| Not applicable \| |
| tp2_h5b | b) Your employer | radio (Matrix), Required   \| 1 \| 1 Very low confidence \| \| --- \| --- \| \| 2 \| 2 \| \| 3 \| 3 \| \| 4 \| 4 \| \| 5 \| 5 \| \| 6 \| 6 \| \| 7 \| 7 Very high confidence \| \| 8 \| Not applicable \| |
| tp2_h5c | c) Local hospitals | radio (Matrix), Required   \| 1 \| 1 Very low confidence \| \| --- \| --- \| \| 2 \| 2 \| \| 3 \| 3 \| \| 4 \| 4 \| \| 5 \| 5 \| \| 6 \| 6 \| \| 7 \| 7 Very high confidence \| \| 8 \| Not applicable \| |
| tp2_h5d | d) UF Health | radio (Matrix), Required   \| 1 \| 1 Very low confidence \| \| --- \| --- \| \| 2 \| 2 \| \| 3 \| 3 \| \| 4 \| 4 \| \| 5 \| 5 \| \| 6 \| 6 \| \| 7 \| 7 Very high confidence \| \| 8 \| Not applicable \| |
| tp2_h5e | e) Florida Department of Health | radio (Matrix), Required   \| 1 \| 1 Very low confidence \| \| --- \| --- \| \| 2 \| 2 \| \| 3 \| 3 \| \| 4 \| 4 \| \| 5 \| 5 \| \| 6 \| 6 \| \| 7 \| 7 Very high confidence \| \| 8 \| Not applicable \| |
| tp2_h5f | f) Centers for Disease Control and Prevention | radio (Matrix), Required   \| 1 \| 1 Very low confidence \| \| --- \| --- \| \| 2 \| 2 \| \| 3 \| 3 \| \| 4 \| 4 \| \| 5 \| 5 \| \| 6 \| 6 \| \| 7 \| 7 Very high confidence \| \| 8 \| Not applicable \| |
| tp2_h5g | g) Public schools | radio (Matrix), Required   \| 1 \| 1 Very low confidence \| \| --- \| --- \| \| 2 \| 2 \| \| 3 \| 3 \| \| 4 \| 4 \| \| 5 \| 5 \| \| 6 \| 6 \| \| 7 \| 7 Very high confidence \| \| 8 \| Not applicable \| |
| tp2_h5h | h) Public transportation companies | radio (Matrix), Required   \| 1 \| 1 Very low confidence \| \| --- \| --- \| \| 2 \| 2 \| \| 3 \| 3 \| \| 4 \| 4 \| \| 5 \| 5 \| \| 6 \| 6 \| \| 7 \| 7 Very high confidence \| \| 8 \| Not applicable \| |
| tp2_h5i | i) Police | radio (Matrix), Required   \| 1 \| 1 Very low confidence \| \| --- \| --- \| \| 2 \| 2 \| \| 3 \| 3 \| \| 4 \| 4 \| \| 5 \| 5 \| \| 6 \| 6 \| \| 7 \| 7 Very high confidence \| \| 8 \| Not applicable \| |
| tp2_h5j | j) Church/places of worship | radio (Matrix), Required   \| 1 \| 1 Very low confidence \| \| --- \| --- \| \| 2 \| 2 \| \| 3 \| 3 \| \| 4 \| 4 \| \| 5 \| 5 \| \| 6 \| 6 \| \| 7 \| 7 Very high confidence \| \| 8 \| Not applicable \| |
| tp2_h6text | H6. Answer the questions below whether you agree or disagree. | descriptive |
| tp2_h6a | a) I have a hard time making it through stressful events | radio (Matrix), Required   \| 1 \| 1 Completely disagree \| \| --- \| --- \| \| 2 \| 2 \| \| 3 \| 3 \| \| 4 \| 4 \| \| 5 \| 5 \| \| 6 \| 6 \| \| 7 \| 7 Completely agree \| |
| tp2_h6b | b) It does not take me long to recover from a stressful event | radio (Matrix), Required   \| 1 \| 1 Completely disagree \| \| --- \| --- \| \| 2 \| 2 \| \| 3 \| 3 \| \| 4 \| 4 \| \| 5 \| 5 \| \| 6 \| 6 \| \| 7 \| 7 Completely agree \| |
| tp2_h6c | c) It is hard for me to snap back when something bad happens | radio (Matrix), Required   \| 1 \| 1 Completely disagree \| \| --- \| --- \| \| 2 \| 2 \| \| 3 \| 3 \| \| 4 \| 4 \| \| 5 \| 5 \| \| 6 \| 6 \| \| 7 \| 7 Completely agree \| |
| tp2_h7text | H7. Please now give your opinion on the following statements: | descriptive |
| tp2_h7a | a) If a COVID-19 vaccine becomes available and is recommended for me, I would get it. | radio (Matrix), Required   \| 1 \| 1 Completely disagree \| \| --- \| --- \| \| 2 \| 2 \| \| 3 \| 3 \| \| 4 \| 4 \| \| 5 \| 5 \| \| 6 \| 6 \| \| 7 \| 7 Completely agree \| |
| tp2_h7b | b) In the event of an outbreak it is appropriate to avoid certain people on the basis of their race/ethnicity and associated risk | radio (Matrix), Required   \| 1 \| 1 Completely disagree \| \| --- \| --- \| \| 2 \| 2 \| \| 3 \| 3 \| \| 4 \| 4 \| \| 5 \| 5 \| \| 6 \| 6 \| \| 7 \| 7 Completely agree \| |
| tp2_h7c | c) I think that the restrictions currently being implemented are greatly exaggerated | radio (Matrix), Required   \| 1 \| 1 Completely disagree \| \| --- \| --- \| \| 2 \| 2 \| \| 3 \| 3 \| \| 4 \| 4 \| \| 5 \| 5 \| \| 6 \| 6 \| \| 7 \| 7 Completely agree \| |
| tp2_h7d | d) The government should be allowed to force people into self-isolation if they have been in contact with someone who was infected with coronavirus. | radio (Matrix), Required   \| 1 \| 1 Completely disagree \| \| --- \| --- \| \| 2 \| 2 \| \| 3 \| 3 \| \| 4 \| 4 \| \| 5 \| 5 \| \| 6 \| 6 \| \| 7 \| 7 Completely agree \| |
| tp2_h7e | e) More tests for coronavirus infection should be carried out in the population | radio (Matrix), Required   \| 1 \| 1 Completely disagree \| \| --- \| --- \| \| 2 \| 2 \| \| 3 \| 3 \| \| 4 \| 4 \| \| 5 \| 5 \| \| 6 \| 6 \| \| 7 \| 7 Completely agree \| |
| tp2_h7f | f) I am worried that the pandemic will have economic consequences for me in the future | radio (Matrix), Required   \| 1 \| 1 Completely disagree \| \| --- \| --- \| \| 2 \| 2 \| \| 3 \| 3 \| \| 4 \| 4 \| \| 5 \| 5 \| \| 6 \| 6 \| \| 7 \| 7 Completely agree \| |
| tp2_h8text | H8. Please indicate if you have: | descriptive |
| tp2_h8a | a) Avoided people based on their ethnicity who I thought might infect me | radio (Matrix), Required   \| 1 \| Yes \| \| --- \| --- \| \| 0 \| No \| \| 2 \| Not applicable \| |
| tp2_h8b | b) Exercised less than I did before the pandemic | radio (Matrix), Required   \| 1 \| Yes \| \| --- \| --- \| \| 0 \| No \| \| 2 \| Not applicable \| |
| tp2_h8c | c) Drank more alcohol than I did before the pandemic | radio (Matrix), Required   \| 1 \| Yes \| \| --- \| --- \| \| 0 \| No \| \| 2 \| Not applicable \| |
| tp2_h8d | d) Ate more unhealthy food than I did before the pandemic | radio (Matrix), Required   \| 1 \| Yes \| \| --- \| --- \| \| 0 \| No \| \| 2 \| Not applicable \| |
| tp2_h8e | e) Smoked more than I did before the pandemic | radio (Matrix), Required   \| 1 \| Yes \| \| --- \| --- \| \| 0 \| No \| \| 2 \| Not applicable \| |
| tp2_h8f | f) Postponed vaccination for myself or my child | radio (Matrix), Required   \| 1 \| Yes \| \| --- \| --- \| \| 0 \| No \| \| 2 \| Not applicable \| |
| tp2_h8g | g) Avoided going to the doctor for a non-COVID-19-related problem | radio (Matrix), Required   \| 1 \| Yes \| \| --- \| --- \| \| 0 \| No \| \| 2 \| Not applicable \| |
| tp2_h8h | h) Bought drugs that I heard are good for treating COVID-19 | radio (Matrix), Required   \| 1 \| Yes \| \| --- \| --- \| \| 0 \| No \| \| 2 \| Not applicable \| |
| ***tp2_work, tp2_work_status, tp2_work_med, tp2_lostincome* remain the same as for tp1 (only difference being the timepoint). See tp1 table for questions asked to participants and the possible answers/code.** | | |
| tp2_remote_inperson | Q01. Is your child currently enrolled in in-person learning, where s/he is physically on PKY's campus in-person?  *Note: your child may have been asked to isolate at home is s/he is a potential contact. Please indicate here the choice you indicated to PKY and where your child is registered as a student.* | yesno, Required   \| 1 \| Yes \| \| --- \| --- \| \| 0 \| No \| |
| tp2_sports | Q02. Is your child playing sports this fall? | radio, Required   \| 1 \| Yes \| \| --- \| --- \| \| 0 \| No \| \| 2 \| Haven't decided \| |
| tp2_band | Q03. Is your child in the school band? | radio, Required   \| 1 \| Yes \| \| --- \| --- \| \| 0 \| No \| \| 2 \| Haven't decided \| |
| tp2_band_inperson  Show the field ONLY if:  [tp2_band] = '1' | In person? | yesno, Required   \| 1 \| Yes \| \| --- \| --- \| \| 0 \| No \| |
| ***tp2_flu_vacc, tp2_flu_vacc_19, tp2_flu_vacc_method*** **remain the same as for tp1 (only difference being the timepoint). See tp1 table for questions asked to participants and the possible answers/code.** | | |
| tp2_hh_number | 1. How many persons live in the child's household(s) in the period since the last questionnaire (in cases where the child lives between more than one household, please combine the number in both households) | text (integer), Required |
| tp2_hh_child | 1.A Between how many households does the child live? | text (integer), Required |
| tp2_hh_illness | 2. Has any member of the child's collective household had COVID-19 with or without lab confirmed test, or had a COVID-19-like viral syndrome of unknown cause? | yesno, Required   \| 1 \| Yes \| \| --- \| --- \| \| 0 \| No \| |
| tp2_fam_death | 4. Has anyone in the family died from COVID-19? | yesno, Required   \| 1 \| Yes \| \| --- \| --- \| \| 0 \| No \| |
| ***tp2_cscm1 to tp2_cscm16 and tp2_13cscm2 to tp2_13cscm17* remain the same as for tp1 (only difference being the timepoint). See tp1 table for questions asked to participants and the possible answers/code.** | | |

### TP3 Questionnaire

Most of the TP3 questionnaire remained the same as TP2 except for two main changes.

1. The addition of a few variables. Below are the few variables that were added. All others are as they appear in TP1 and TP2, but with the change in the variable name to indicate “tp3”.
2. The removal of some variables

**Note: Most variables remained from TP2 to TP3, and these were unchanged. Variables where either added or deleted, but not modified.**

tp3_skip: Variable to indicate (if value=1) that parent had already filled out the household portion of the questionnaire for another participant.

|  | Variable name | Question asked participants | Possible answers/Code |
| --- | --- | --- | --- |
|  | tp3_lostincome_2  Show the field ONLY if:  [tp3_skip3] <> '1' and [tp3_lostincome] = '1' | Is that person still out of work? | yesno, Required   \| 1 \| Yes \| \| --- \| --- \| \| 0 \| No \| |
|  | tp3_ethnicity | Is your child Hispanic or Latino? (Check only one) | radio, Required   \| 1 \| Yes, my student is Hispanic/Latino -- a person of Cuban, Mexican, Puerto Rican, South or Central American, or other Hispanic culture or origin, regardless of race \| \| --- \| --- \| \| 0 \| No, my student is not Hispanic/Latino \| |
|  | tp3_race0 | Please check ALL racial descriptors that apply to your child. | American Indian or Alaska Native -- a person having origins in any of the original peoples of North and South America (including Central America) and who maintains tribal affiliation or community attachment |
|  | tp3_race1 | Please check ALL racial descriptors that apply to your child. | Asian -- a person having origins in any of the original peoples of the Far East, Southeast Asia, or the Indian subcontinent, e.g., Cambodia, China, India, Japan, Korea, Malaysia, Pakistan, the Philippine Islands, Thailand, and Vietnam |
|  | tp3_race2 | Please check ALL racial descriptors that apply to your child. | Black or African American -- a person having origins in any of the black racial groups of Africa |
|  | tp3_race3 | Please check ALL racial descriptors that apply to your child. | Native Hawaiian or Other Pacific Islander -- a person having origins in any of the original peoples of Hawaii, Guam, Samoa, or other Pacific Islands |
|  | tp3_race4 | Please check ALL racial descriptors that apply to your child. | White -- a person having origins in any of the original peoples of Europe, the Middle East, or North Africa |
|  | tp3_hh_vaccine | 2. Considering all of these household members, how many have been fully vaccinated for COVID-19 more than 2 weeks ago? (Fully vaccinated means that 14 days have passed after the last dose of the vaccine. If the vaccine was from Pfizer or Moderna, this would be 14 days after the second dose, if it was from Johnson & Johnson, this is 14 days after first and only dose) | text (integer), Required |
|  | tp3_hh_refvax | 3. Has anyone in the household been offered a COVID-19 vaccine and refused, or is eligible to sign up to get a COVID-19 vaccine and refused? | yesno, Required   \| 1 \| Yes \| \| --- \| --- \| \| 0 \| No \| |
|  | tp3_hh_refvaxwhy | Why? | text, Required |
|  | tp3_agever | How many years old is the [student name] | Text, integer. |
|  | baseline_mh | Binary variable determining if the student was identified by the study as being previously at risk (automatically generated variable). | 1= At previous risk  0= Had not been identified as at risk |

### HH Contact Questions (TP3)

| tp3_hhcontact_dob | Date of birth (for HH contacts only) | Date MM/DD/YYYY |
| --- | --- | --- |
| tp3_hhcontact_age | Age of household contact | Number |
| tp3_hhcontact_ethnicity | 6. Do you (household contact) identify as Hispanic or Latino? (Check only one) | radio, Required   \| 1 \| Yes, household contact is Hispanic/Latino -- a person of Cuban, Mexican, Puerto Rican, South or Central American, or other Hispanic culture or origin, regardless of race \| \| --- \| --- \| \| 0 \| No, household contact is not Hispanic/Latino \| |
| tp3_hhcontact_race | 7. Please check ALL racial descriptors that apply to you (household contact). | checkbox, Required   \| 1 \| tp3_hhcontact_race0 \| American Indian or Alaska Native -- a person having origins in any of the original peoples of North and South America (including Central America) and who maintains tribal affiliation or community attachment \| \| --- \| --- \| --- \| \| 2 \| tp3_hhcontact_race1 \| Asian -- a person having origins in any of the original peoples of the Far East, Southeast Asia, or the Indian subcontinent, e.g., Cambodia, China, India, Japan, Korea, Malaysia, Pakistan, the Philippine Islands, Thailand, and Vietnam \| \| 3 \| tp3_hhcontact_race2 \| Black or African American -- a person having origins in any of the black racial groups of Africa \| \| 4 \| tp3_hhcontact_race3 \| Native Hawaiian or Other Pacific Islander -- a person having origins in any of the original peoples of Hawaii, Guam, Samoa, or other Pacific Islands \| \| 5 \| tp3_hhcontact_race4 \| White -- a person having origins in any of the original peoples of Europe, the Middle East, or North Africa \| |
| tp3_hhcontact_schooling | 8. How many years of schooling do you (household contact) have (e.g. high school = 12; college =16; etc.) | text (integer, Min: 0, Max: 30), Required |
| tp3_hhcontact_positive | 9. Have you (household contact) tested positive for SARS-CoV-2, been told by a health care provider that you were positive for COVID-19, or had positive antibody test for SARS-CoV-2? | radio, Required   \| 1 \| Yes \| \| --- \| --- \| \| 0 \| No \| \| 2 \| I don't know/Unsure \| |
| tp3_hhcontact_vaccine | 10. Have you (household contact) been fully vaccinated against COVID-19? (For Pfizer and Moderna vaccines full vaccination requires two doses, for Johnson and Johnson one dose) | radio, Required   \| 0 \| Yes, I am fully vaccinated against COVID-19 \| \| --- \| --- \| \| 1 \| No, I have only received the first vaccine in a two dose series against COVID-19 \| \| 2 \| No, I have not yet received any COVID-19 vaccine \| \| 3 \| Other \| |
| tp3_hhcontact_vaccine_other  Show the field ONLY if:  [tp3_hhcontact_vaccine] = '3' | Please specify: | text, Required |
| tp3_hhcontact_which_vaccine  Show the field ONLY if:  [tp3_hhcontact_vaccine] = '0' | 11. If yes, Which vaccine did you receive? | radio, Required   \| 1 \| Pfizer \| \| --- \| --- \| \| 2 \| Moderna \| \| 3 \| Johnson & Johnson \| \| 4 \| Other: \| |
| tp3_hhcontact_which_vaccine_other  Show the field ONLY if:  [tp3_hhcontact_which_vaccine] = '4' | If other, which ___? | text, Required |
| tp3_hhcontact_vaccinedate  Show the field ONLY if:  [tp3_hhcontact_vaccine] = '0' | 12. Do you know the date of your final vaccine dose? | yesno, Required   \| 1 \| Yes \| \| --- \| --- \| \| 0 \| No \| |
| tp3_hhcontact_vaccinedate_no  Show the field ONLY if:  [tp3_hhcontact_vaccinedate] = '0' | In what month did you receive your final dose? | radio, Required   \| 12 \| December \| \| --- \| --- \| \| 1 \| January \| \| 2 \| February \| \| 3 \| March \| \| 4 \| April \| \| 0 \| I don't know \| |

# 5.Supplemental Descriptors

These data are currently stored at the Dropbox servers under the University of Florida business account to which the PI’s have access. The data is also stored separately at the Clinical and Translational Science Institute at the University of Florida servers. These data will be published in an online repository in October of 2022. A link to these data will be added here once it is stored.

Two publications using these data has been published so far.

- McKune, S. L., Acosta, D., Diaz, N., Brittain, K., Beaulieu, D. J., Maurelli, A. T., & Nelson, E. J. (2021). Psychosocial health of school-aged children during the initial COVID-19 safer-at-home school mandates in Florida: a cross-sectional study. BMC public health, 21(1), 1-11.
- Acosta, D., Fujii, Y., Joyce-Beaulieu, D., Jacobs, K. D., Maurelli, A. T., Nelson, E. J., & McKune, S. L. (2021). Psychosocial health of K-12 students engaged in emergency remote education and in-person schooling: a cross-sectional study. *International journal of environmental research and public health*, *18*(16), 8564.

A third and final publication by the authors of the study using this data is forthcoming, once it is published, it will be linked here.
