# Supplementary material for: The infected and the affected: A longitudinal study of the impact of the COVID-19 pandemic on schoolchildren in Florida
Source: Front Public Health. 2023 Mar 8;11:1003923. doi: 10.3389/fpubh.2023.1003923 (PMC10030597; doi:10.3389/fpubh.2023.1003923)
Supplement: Supplementary file 2 [file Table_2.DOCX]

| **Household Contacts^1^** | | | | | |
| --- | --- | --- | --- | --- | --- |
|  |  |  |  | | |
| PCR | TP1 n=96 | TP2 n=158 | TP3 n=108 | | |
| Positive | 0 (0%) | 0 (0%) | 0 (0%) | | |
| Negative | 96 (100%) | 158 (100%) | 108 (100%) | | |
|  |  |  |  | | |
| Serology | TP1 n=94 | TP2 n=153 | TP3 n=105 | | |
|  |  |  | Fully vaccinated n=65 | Partially vaccinated n=27 | Not vaccinated n=13 |
| Positive | 1 (1.1%) | 6 (3.9%) | 62(95.4%) | 24(88.9%) | 2(15.4%) |
| Negative | 91 (96.8%) | 146 (95.4%) | 2(3.1%) | 2(7.4%) | 11(84.6%) |
| Indeterminate | 2 (2.1%) | 1 (0.7%) | 1(1.5%) | 1(3.7%) | 0 |
