# Supplementary material for: The infected and the affected: A longitudinal study of the impact of the COVID-19 pandemic on schoolchildren in Florida
Source: Front Public Health. 2023 Mar 8;11:1003923. doi: 10.3389/fpubh.2023.1003923 (PMC10030597; doi:10.3389/fpubh.2023.1003923)
Supplement: Supplementary file 3 [file Table_3.DOCX]

**Supplemental Methods Files**

**Outcome Variables**

From the questionnaire, the following items were used to build the variables for symptoms of anxiety, depression, and OCD. If a participant answered with 3, or higher, in the 5-point Likert scale for any of the items in the variables, they were considered “At Risk”.

The 5-point Likert Scale for children under 13 was: Never (1), A little (2), Sometimes (3), A lot (4), or Always/constantly (5). For children over 13: Never (1), Occasionally (2), Half the Time(3) , Often(4) , Always (5)

| Variable | Items |
| --- | --- |
| Depression symptoms for children 13 and younger | I feel hopeless and sad (about the virus) |
|  | I have trouble eating or sleeping |
|  | I find myself crying a lot |
|  | I have a stomachache/headache |
|  | It's hard for me to think a long time |
| Anxiety symptoms for children 13 and younger | I have trouble eating or sleeping |
|  | I feel worried or nervous (about the virus) |
|  | It is hard to stop my thoughts (about the virus) |
|  | I cannot stop worrying (about the virus) |
|  | It's hard for me to think a long time |
| OCD symptoms for children 13 and younger | I feel worried or nervous (about the virus) |
|  | It is hard to stop my thoughts (about the virus) |
|  | I cannot stop worrying (about the virus) |
|  | I am very scared of getting dirty |
|  | I have to wash my hands, over and over to feel better |
| Depression symptoms for children Over 13 | Sadness, feeling down, low mood, feeling fatigued |
|  | Feelings of hopelessness, worthlessness, emptiness, or not being a good person |
|  | Decreased pleasure from things that used to be fun, feeling that life is not much fun |
|  | Being easily annoyed or irritable, feelings of dread like something awful might happen |
| Anxiety symptoms for children Over 13 | Feeling worried, nervous, panicky, tense, keyed-up |
|  | Not being able to stop worrying or controlling your worry |
|  | Being easily annoyed or irritable, feelings of dread like something awful might happen |
|  | Felt a racing heart, shaky sweaty, or had trouble breathing |
| OCD Over symptoms for children 13 | Not being able to stop worrying or controlling your worry |
|  | Constant thoughts about avoiding germs |
|  | Fixation with washing your hands throughout the day |
|  | Sudden moments of fear or terror because you couldn't get rid of the germs |

KAP Scores

**Knowledge questions (all used to calculate the Knowledge score, range 0-16)**

**For the following statements, please indicate if True or False.**

K1. The main clinical symptoms of COVID-19 are fever, fatigue, dry cough, and muscle aches.

K2. Unlike the common cold, stuffy nose, runny nose, and sneezing are less common in persons infected with the COVID-19 virus.

K3. There currently is no effective cure for COVID-19, but early symptomatic and supportive treatment can help most patients recover from the infection.

K4. Antibiotics can be used to treat COVID-19

K5. Not all persons with COVID-19 will develop to severe cases.

K6. People of all racial and ethnic groups can become infected with the COVID-19 virus.

K7. Most people who are infected with the COVID-19 virus recover from it

K8. Handwashing can help reduce transmission of the COVID-19 virus.

K9. Persons with COVID-19 cannot pass the virus to others if they do not have symptoms.

K10.The COVID-19 virus spreads via respiratory droplets from infected individuals.

K11. People can wear general medical masks to prevent infection by the COVID-19 virus.

K12. It is not necessary for children and young adults to take measures to prevent infection by the COVID-19 virus.

K13. Isolation and treatment of people who are infected with the COVID-19 virus are effective ways to reduce the spread of the virus.

K14. People who have contact with someone infected with the COVID-19 virus should be immediately isolated in a proper place for 14 days.

K15. People with a strong immune system will not get infected with COVID-19

K16. To prevent the infection by COVID-19, individuals should avoid going to crowded places such as restaurants, bars, concerts, etc.

**Attitudes about COVID-19**

**For the following statements, please indicate on a scale of 1-5, with 1 being strongly disagree and 5 being strongly agree. The following items were used to calculate the Attitude score: A1, A2, A3, A4, A5, A6, A7, A8, A12, and A14. Answers of 4 and 5 for any of these items were considered protective, and counted as one point Range 0-10)**

1 – Strongly disagree

2 – Disagree

3 – Neither agree nor disagree

4 – Agree

5 – Strongly agree

A1. I am worried about getting infected with the COVID-19 virus.

A2. I feel confident I can prevent myself and my family from becoming infected with the COVID-19 virus.

A3. I know what actions to take to prevent myself and my family from becoming infected with the COVID-19 virus.

A4. I support CDC imposed guidelines for those who are infected with the COVID-19 virus.

A5. I support/would support city and state-imposed regulations to protect the public (e.g. business closures, mask wearing, park closures, beach closures, etc.)

A6. I support postponing or canceling mass gatherings such as concerts, festivals, and sporting events.

A7. I support closure of K-12 schools if any student, staff member, or teacher is found to have COVID-19.

A8. If I were exposed to and could possibly be infected with the COVID-19 virus, I would be willing to quarantine myself at home for 2 weeks until I was sure I was not infected, in order to prevent others from getting COVID-19 from me.

A9. I support K-12 schools re-opening in person instruction this fall.

A10. I am comfortable going out in public as long as I am in open spaces.

A11. I am comfortable going into essential public spaces as necessary, such as grocery stores, pharmacies, etc.

A12. I am comfortable going into public retail spaces, such as department stores, restaurants, etc.

A13. I think that the US government is handling the COVID-19 health crisis well.

A 14. I support mask mandates in closed-in

**Practices about COVID-19**

**All answers are Yes/No, and all items are used to calculate the Practice Scores. When participants responded “Yes” this was considered a protective practice, and counted for one point (Range=0-8).**

P1. In recent days, I am washing my hands with soap and water more often than normal?

P2. In recent days, I am using more disinfectants, such as hand sanitizers and cloth wipes?

P3. In recent days, I am avoiding shaking hands or other physical contact with others outside my home?

P4. In recent days, I have adhered to social distancing guidelines, such as avoiding meetings of more than 10 people and keeping a distance of 6 feet apart?

P5. In recent days, I have bought larger amounts of staple foods (flour, sugar, pasta, rice, canned food) than normal?

P6. I am avoiding going into any crowded place.

P7. I wear a mask when leaving home and entering an indoor area with other people.

P8. In the past 7 days I have stayed at home or worked from home rather than going into work.
